# PEGylated liposomal metformin overcomes pharmacokinetic barriers to trigger potent mitochondrial disruption and cell cycle arrest in hepatocellular carcinoma

**DOI:** 10.1038/s41598-025-13280-0

**Published:** 2025-08-07

**Authors:** Zeinab A. Elzanaty, Medhat W. Shafaa, Seifeldin Elabed, Mohamed M. Omran

**Affiliations:** 1 Biochemistry Division, Chemistry Department, Faculty of Science, Helwan University, Cairo, Egypt; 2 Medical Biophysics Division, Physics Department, Faculty of Science, Helwan University, Cairo, Egypt

**Keywords:** PEGylated liposomes, Metformin nanocarriers, Hepatocellular carcinoma, Apoptosis, Cell cycle arrest, Molecular dynamics simulation, MM-PBSA

## Abstract

This study presents a comprehensive experimental and computational evaluation of PEGylated liposomal metformin as a nanocarrier-based therapeutic strategy for hepatocellular carcinoma (HCC). Liposomal formulations were prepared via thin-film hydration, yielding spherical, well-dispersed vesicles with high encapsulation efficiency (> 90%) and a mean hydrodynamic diameter of 177.2 ± 30.2 nm. PEGylation and metformin loading induced significant physicochemical alterations, as confirmed by differential scanning calorimetry and FTIR spectroscopy, reflecting increased bilayer fluidity and headgroup interactions. Cytotoxicity assays revealed a substantial enhancement in antitumor potency: PEGylated liposomal metformin reduced the IC₅₀ against HepG2 cells to 118.76 μg/mL compared to 2392.81 μg/mL for free metformin—representing a > 20-fold improvement. In Vero cells, IC₅₀ values were 137.13 μg/mL and 2113.86 μg/mL, respectively, yielding a selectivity index of 1.15. Apoptosis analysis demonstrated increased early and late apoptotic populations, with PEGylated formulations inducing total apoptosis rates of 20.67% in HepG2 cells. Cell cycle profiling revealed marked G₀/G₁ arrest, with 78.12% accumulation versus 58.21% in untreated controls. DNA fragmentation analysis via comet assay further supported elevated genotoxic effects in cancer cells. Molecular docking and 100 ns molecular dynamics simulations confirmed stable binding of metformin to mitochondrial Complex I and CDK4/cyclin D3, with a total MM-PBSA binding energy of − 27.33 kcal/mol in the CDK4 complex. These findings demonstrate that PEGylated liposomal encapsulation substantially enhances the cytotoxic profile of metformin, supporting its advancement as a targeted nanotherapeutic candidate for HCC.

## Introduction

Hepatocellular carcinoma (HCC) remains a formidable global health challenge as the most common primary liver malignancy and third-leading cause of cancer mortality worldwide, with over 900,000 new cases and 830,000 deaths annually^1,2^. The disease exhibits striking geographical disparities, disproportionately burdening regions with endemic hepatitis B/C infections, dietary aflatoxin exposure, and a rising prevalence of metabolic syndrome^2,3^. Despite advances in locoregional therapies and systemic agents (sorafenib, lenvatinib, immune checkpoint inhibitors), 5-year survival for advanced HCC remains below 20% due to high recurrence rates, therapeutic resistance, and dose-limiting toxicity^4,5^. These limitations underscore the urgent need for innovative treatment strategies with improved tumor selectivity.

Metformin (1,1-dimethylbiguanide)—a cationic biguanide first synthesized in 1922 and clinically established for type 2 diabetes—has emerged as a promising anticancer candidate due to its distinctive chemical architecture. The planar arrangement of protonatable guanidine groups facilitates Organic Cation Transporter (OCT)-dependent uptake and mitochondrial accumulation^6,7^, enabling inhibition of respiratory complex I^8^. This suppresses ATP production, activates AMPK, and downregulates mTOR signaling—a pathway dysregulated in 40–50% of HCC cases^9,10^. Epidemiological studies reveal a consistent 30–40% reduction in HCC incidence among diabetic patients using metformin versus other glucose-lowering agents^11^. Preclinical evidence demonstrates direct antitumor effects including G₀/G₁ arrest via cyclin D1 suppression^12^ and epigenetic modulation through TET2 activation^13^. Its metabolic influence also intersects with pathways like one-carbon metabolism, where enzymes such as serine hydroxymethyltransferase 2 (SHMT2) are recognized as crucial therapeutic targets^14^. Furthermore, metformin shows strong potential to synergize with established kinase inhibitors like sorafenib, significantly suppressing tumor cell proliferation compared to monotherapy in preclinical models^15^.

Despite compelling mechanisms, clinical translation faces significant pharmacokinetic barriers. Metformin’s high hydrophilicity (logP =  − 1.43) limits membrane permeability and oral bioavailability (< 60%), while rapid renal clearance (t1/2≈6.5 h) necessitates high daily doses (1–2 g) that cause gastrointestinal toxicity and constrain anticancer efficacy^16^. To overcome these limitations, we engineered PEGylated liposomes leveraging metformin’s cationic nature for electrostatic encapsulation within soy lecithin (phosphatidylcholine)/cholesterol bilayers (55:40 molar ratio) with 5 mol% DSPE-PEG₂₀₀₀. This nanotechnology approach exploits the Enhanced Permeability and Retention (EPR) effect—optimal for nanoparticles of 100–150 nm—to enhance tumor accumulation while minimizing systemic exposure^17,18^. PEGylation further extends circulation half-life by reducing opsonization and reticuloendothelial clearance^19^.

This study integrates experimental and computational strategies to: (1) characterize the physicochemical properties of PEGylated liposomal metformin, (2) evaluate cytotoxic efficacy in HCC line (HepG2) versus Vero kidney epithelial cells (selected as a non-tumorigenic control for standardized toxicity assessment^20^), and (3) elucidate molecular interactions with mitochondrial complex I and cell cycle regulators (CDK4/cyclin D3) through docking and dynamics simulations. Our convergent approach addresses fundamental questions about metformin’s polypharmacology while advancing nanoformulation strategies to enhance therapeutic index.

Looking forward, this framework could enable derivative design targeting specific cancer vulnerabilities or combination regimens with immunotherapy—particularly relevant for HCC’s immunosuppressive microenvironment^21^. By bridging medicinal chemistry, nanotechnology, and computational biology, we exemplify a rational paradigm for repurposing established drugs into precision oncology therapeutics.

## Materials and methods

### Chemicals and reagents

All chemicals and reagents used were of analytical grade or higher purity. Metformin (molecular weight: 129.164 g/mol) was procured from EIPICO (Egyptian International Pharmaceutical Industries Co, Egypt). L-α-phosphatidylcholine (Soy Lecithin; molecular weight: 760 g/mol, purity ≥ 97%) was sourced from Carl Roth (Karlsruhe, Germany), while DSPE-PEG₂₀₀₀ and stearylamine were obtained from Sigma-Aldrich (St. Louis, MO, USA). Absolute ethanol (99.9% purity) was purchased from DaeJung Chemicals (Seohaean-ro, Gyeonggi-do, Korea), and phosphate-buffered saline (PBS, pH 7.4) was acquired from CDH (New Delhi, India). Cell culture reagents including DMEM medium, fetal bovine serum (FBS), penicillin–streptomycin, and trypsin–EDTA were procured from Sigma-Aldrich. All solutions were prepared using ultra-pure distilled water (Milli-Q®, 18.2 MΩ·cm), with solvents meeting HPLC-grade standards. Biochemical assay kits for MTT, Annexin V-FITC/PI apoptosis detection, and comet assays were sourced from Thermo Fisher Scientific (Waltham, MA, USA).

**Fig. 1:**
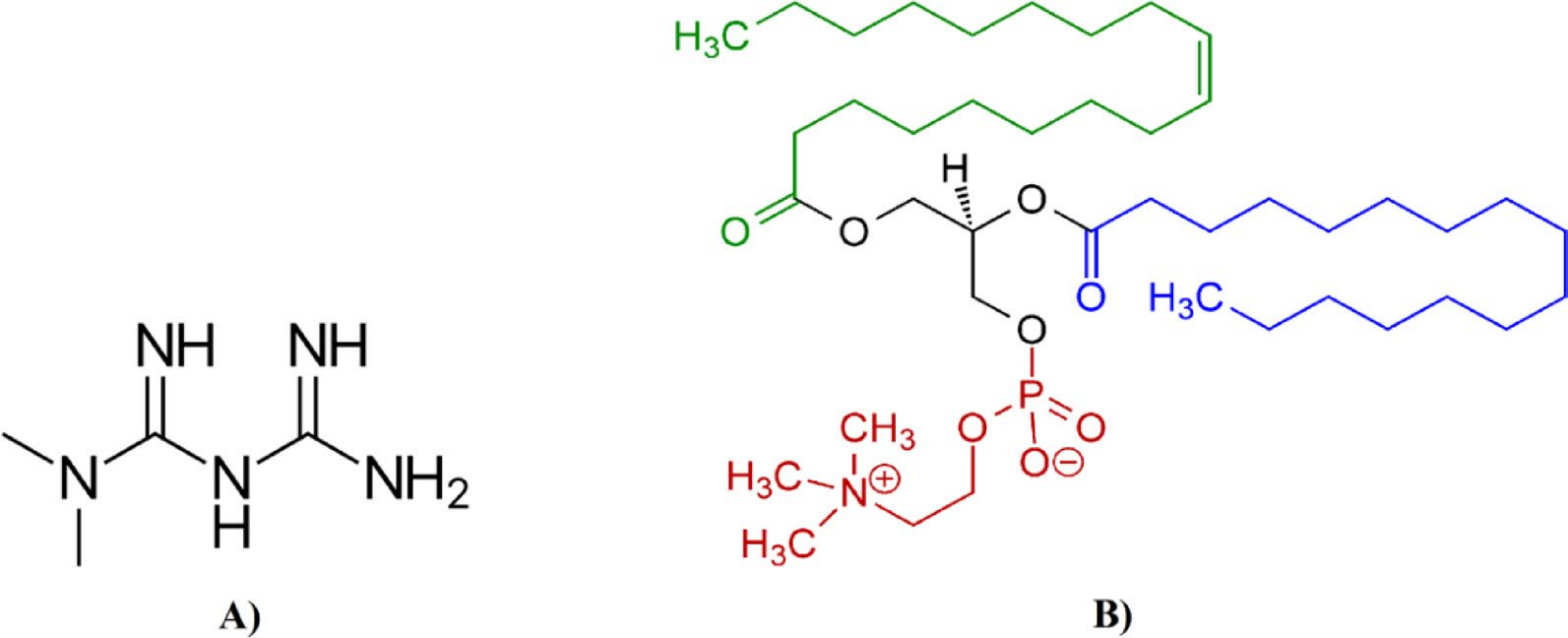
(**A**) The chemical structure of Metformin. (**B**) The schematic chemical structure of L-α-phosphatidylcholine (Soy Lecithin).

The human hepatocellular carcinoma cell line HEPG-2 (HB-8065) and African green monkey kidney epithelial cell line Vero (CCL-81) were obtained from Vacsera Cell Culture Unit (Cairo, Egypt) with authentication performed via short tandem repeat (STR) profiling. HEPG-2 cells, originally derived from a 15-year-old male hepatocellular carcinoma patient, and Vero cells, established from kidney epithelium, were selected as cancer and non-tumorigenic models respectively, consistent with ISO 10,993–5 standards for cytotoxicity screening. Both cell lines were maintained in DMEM supplemented with 10% heat-inactivated FBS, 100 U/mL penicillin, and 100 μg/mL streptomycin at 37 °C in a humidified 5% CO₂ atmosphere. Cells were passaged at 80–90% confluence using 0.25% trypsin–EDTA and routinely monitored for morphological consistency and absence of mycoplasma contamination (Fig. 1).

### Liposome preparation and encapsulation efficiency

Liposomes were prepared using the thin-film hydration method of Bangham^22^ with optimized modifications. Briefly, 100 mg soy lecithin (L-α-phosphatidylcholine, Carl Roth) and 5 mg metformin (EIPICO) were dissolved in 30 ml absolute ethanol (99.9%, DaeJung Chemicals) in a 100 ml round-bottom flask. The mixture was evaporated under reduced pressure at 55 °C using a rotary evaporator (Heidolph, Germany) to form a uniform lipid film. For PEGylated formulations, 40 mg DSPE-PEG₂₀₀₀ (Sigma-Aldrich) was co-dissolved with lecithin, consistent with established protocols maximizing stealth properties at 5–10% w/w lipid^19^. The lipid film was hydrated with 10 ml pre-warmed (55 °C) phosphate-buffered saline (PBS; pH 7.4, CDH Chemicals) under mechanical shaking (200 rpm) for 1 h at 55 °C to form multilamellar vesicles (MLVs). The suspension was flushed with nitrogen and sealed to prevent oxidation. Control liposomes (empty and PEGylated) were prepared identically without metformin.

Non-encapsulated metformin was separated by centrifugation (6000 rpm, 20 min, 4 °C). The supernatant was analyzed at 233 nm using a UV–Vis spectrophotometer (Jasco V-630), with quantification via a metformin calibration curve (0.1–100 μg/ml, R^2^ = 0.999). Encapsulation efficiency was calculated as:

1 $$EE\% = \frac{{Total\; drug\; input\;\left( {{\text{mg}}} \right) - Drug\; in\; supernatant\;\left( {{\text{mg}}} \right)}}{{Total\; drug \;input\;\left( {{\text{mg}}} \right)}} \times 100$$

### Physicochemical characterization

#### Dynamic light scattering (DLS) and zeta potential

Liposome size distribution, polydispersity index (PDI), and zeta potential were analyzed using dynamic light scattering (Nanotrac Wave II, Microtrac, USA). Samples were diluted 1:20 in Tris buffer (10 mM, pH 7.4) and equilibrated at 25 °C for 5 min prior to measurement. Size and PDI were determined via backscatter detection (173° angle) with 30 runs per measurement. Zeta potential was measured using phase analysis light scattering (PALS) with a minimum of 20 replicates. The study evaluated the efficacy of various liposomal formulations, including empty liposomes, metformin-loaded liposomes, and PEGylated metformin liposomes. Results for each formulation were reported as mean ± standard deviation (SD), ensuring statistical clarity in the comparative analysis of their physicochemical properties and potential therapeutic effects.

#### Thermal analysis (DSC)

Thermal transitions were characterized using differential scanning calorimetry (DSC-50, Shimadzu, Japan). Lyophilized samples (5.0 ± 0.1 mg) were sealed in aluminum pans and heated from 25 °C to 100 °C at 3 °C/min under nitrogen purge (50 ml/min). An empty aluminum pan served as reference. Phase transition temperatures (Tm​) were determined from endothermic peak minima using TA-60 software.

#### Fourier-transform infrared (FTIR) spectroscopy

Chemical interactions were analyzed using a Jasco FT/IR-4100 spectrometer. Lyophilized liposomes were mixed with KBr (1:100 ratio) and pressed into transparent discs. Spectra were acquired over 4000–1000 cm⁻^1^ at 4 cm⁻^1^ resolution with 64 scans and 2 mm/s mirror speed. Background correction was performed using pure KBr discs. Peak assignments followed established lipid bilayer band designations^23^.

### In vitro cellular and molecular effects of metformin and its liposomal formulations on hepatocellular carcinoma cells

#### In vitro cytotoxicity assay (MTT)

Cytotoxicity was evaluated against HepG2 and Vero cells using the MTT assay according to established protocols^24^. Following trypsinization and cell counting via Trypan blue exclusion^24^, cells were seeded in 96-well plates at 1 × 10^4^ cells/well in RPMI-1640 medium and allowed to adhere for 24 h. Test compounds (free metformin, empty liposomes, metformin-loaded liposomes, and PEGylated liposomal metformin) were applied in two-fold serial dilutions (100–1200 μg/ml) for 48 h. After incubation, cells were treated with MTT solution (0.5 mg/ml, 50 μl) for 4 h at 37 °C. Formazan crystals were dissolved in DMSO (50 μl), and absorbance measured at 490 nm using a microplate reader (BioTek ELx808). Staurosporine (1 μM) served as the positive control for cytotoxicity. Cell viability was calculated as percentage relative to untreated controls, with IC₅₀ values derived from dose–response curves (four-parameter logistic model)^25^.

Statistical analysis was performed in R using a Type III two-way ANOVA to assess the effects of formulation type, concentration, and their interaction on viability^26,27^. Post-hoc pairwise comparisons were conducted using estimated marginal means (EMMs) with Tukey’s adjustment for multiple comparisons, employing the emmeans package^28^. The model was fitted as a linear model with mean viability as the dependent variable. All experiments were performed in biological triplicate (n = 3) with technical replicates.

#### DNA damage assessment (comet assay)

DNA fragmentation was quantified using the alkaline comet assay according to Singh et al.^29^ with modifications. Briefly, 1 × 10^5^ cells/ml were suspended in 0.5% low-melting-point agarose and layered onto pre-coated slides (0.7% normal agarose base). After solidification, slides were immersed in lysis buffer (2.5 M NaCl, 100 mM EDTA, 10 mM Tris, 1% Triton X-100, 10% DMSO, pH 10) for 1 h at 4 °C. DNA unwinding was performed in alkaline electrophoresis buffer (300 mM NaOH, 1 mM EDTA, pH > 13) for 30 min at 4 °C. Electrophoresis proceeded at 25 V (300 mA) for 30 min, followed by neutralization (0.4 M Tris–HCl, pH 7.5) and staining with ethidium bromide (10 μg/ml). Hydrogen peroxide (100 μM, 30 min) served as the positive control^30^. Comets were visualized under fluorescence microscopy (Olympus IX73, 400 × magnification), with 100 randomly selected comets per slide analyzed using CometScore 2.0 software to determine tail DNA (%), tail length (μm), and olive tail moment^29^.

Triplicate experiments were performed with data expressed as mean ± SD. One-way ANOVA was conducted to assess differences among treatment groups for each dataset separately. Post-hoc comparisons were performed using estimated marginal means (EMMs) with Tukey’s adjustment to control for Type I error. Assumptions of normality and homoscedasticity were verified using standard diagnostic plots prior to conducting ANOVA analyses^27,31^. A *p*-value < 0.05 was considered statistically significant.

#### Apoptosis detection by flow cytometry

Apoptosis was quantified using Annexin V-FITC/PI dual staining per manufacturer protocols (Thermo Fisher Scientific). HepG2 and Vero cells (1 × 10⁶ cells) were treated with IC₅₀ concentrations of test compounds for 48 h. After trypsinization, cells were washed with ice-cold PBS and resuspended in binding buffer. Cells were stained with Annexin V-FITC (5 μl) and PI (10 μl) for 15 min in darkness. Staurosporine (1 μM, 6 h) served as the positive control. Samples were analyzed within 1 h using a BD FACSCalibur™ flow cytometer with CellQuest Pro software. A minimum of 10,000 events were recorded per sample, with populations classified as viable (Annexin V⁻/PI⁻), early apoptotic (Annexin V⁺/PI⁻), late apoptotic (Annexin V⁺/PI⁺), or necrotic (Annexin V⁻/PI⁺). Data analysis utilized FlowJo 10.8 software with gating based on unstained and single-stained controls.

Statistical analysis was performed using triplicate samples (n = 3) with Type III one-way ANOVA^26^ followed by estimated marginal means (EMMs) analysis with Tukey-adjusted pairwise comparisons^28^.

#### Cell cycle analysis

Cell cycle distribution was assessed via propidium iodide (PI) DNA staining. Treated cells (IC₅₀ concentrations, 48 h) were fixed in 70% ethanol at 4 °C overnight. After PBS washing, cells were treated with RNase A (100 μg/ml) at 37 °C for 30 min and stained with PI (50 μg/ml) for 15 min in darkness. Hydroxyurea (2 mM, 24 h) served as the S-phase arrest positive control. Cell suspensions were filtered through 40 μm nylon mesh and analyzed on a BD FACSCalibur™ flow cytometer. DNA content histograms were evaluated using ModFit LT 5.0 software with Goodness-of-Fit criteria (χ^2^ < 3) to quantify G₀/G₁, S, and G₂/M phase distributions.

Statistical analysis employed triplicate samples (n = 3) analyzed through Type III one-way ANOVA^26^. Post-hoc comparisons were conducted using estimated marginal means (EMMs) with 95% confidence intervals and Tukey’s adjustment to determine treatment effects on each cell cycle phase^28^.

### In silico study

#### In silico pharmacokinetic analysis

In silico pharmacokinetic (PK) predictions were conducted to evaluate the absorption, distribution, metabolism, excretion, and toxicity (ADME/T) properties of metformin (PubChem CID: 4091), sorafenib (PubChem CID: 216,239), and lenvatinib (PubChem CID: 9823820) for their potential application in hepatocellular carcinoma (HCC) therapy. Computational tools were selected based on their robust predictive capabilities utilizing graph-based signatures and molecular descriptors. ADMETlab 3.0 was employed to provide comprehensive insights into pharmacokinetic parameters^32^.

Key PK parameters included lipophilicity (log P), topological polar surface area (TPSA), transporter interactions (OATP1B1, OATP1B3, P-gp, BCRP), enzyme inhibition probabilities (BSEP, CYP3A4), hERG blockade probability, and blood–brain barrier (BBB) permeation probability. These parameters were selected for their relevance to HCC pharmacology, particularly in assessing hepatic targeting, drug safety, and potential drug-drug interactions. Predicted values were systematically compared to identify differences in pharmacokinetic profiles and treatment suitability.

#### Protein structure and function analysis

Target proteins involved in HCC progression were analyzed using InterProScan for structural and functional identification. The sequences of mitochondrial Complex I (PDB ID: 5XTD), CDK4/cyclin D3 complex (PDB ID: 3G33), and Bcl-2 (PDB ID: 1G5M) were classified into families, with domain prediction and important site identification^33^. This analysis provided critical insights into protein functional roles and guided binding site selection for molecular docking simulations. PyMOL 3.1.1 was employed for protein structural annotation and visualization of docked poses, facilitating identification of key binding residues and interaction interfaces^34^.

#### Molecular docking simulations

Molecular docking was performed using GNINA 1.3, a deep learning-based tool known for accurate ligand pose prediction and binding affinity assessment^35^. Three-dimensional protein structures were retrieved from the Protein Data Bank (PDB) and prepared by removing water molecules and adding polar hydrogen atoms. Binding sites were defined based on InterProScan domain information: the ubiquinone-binding pocket for mitochondrial Complex I, ATP-binding cleft for CDK4/cyclin D3 complex, and BH3-binding groove for Bcl-2.

Metformin was docked into these sites using GNINA 1.3 default settings. Resulting poses were assessed based on binding affinity scores and visual inspection to confirm plausible interactions. The highest-ranked pose for each target was selected for further analysis. Detailed 2D interaction diagrams were generated using Discovery Studio 2025, illustrating hydrogen bonds, salt bridges, and hydrophobic contacts^36^. PLIP (Protein–Ligand Interaction Profiler) provided complementary 3D visualization, offering comprehensive binding mode analysis^37^.

#### Molecular dynamics simulation and MM-PBSA analysis

Molecular dynamics (MD) simulations and MM-PBSA analyses were conducted to investigate the dynamic behavior and binding stability of metformin (PubChem CID: 4091) with its key targets: the CDK4/cyclin D3 complex (PDB ID: 3G33) and mammalian respiratory Complex I (PDB ID: 5XTD). While both targets demonstrated moderate binding affinities for metformin, interactions with the Bcl-2 structure (PDB ID: 1BXL) were relatively weak, rendering it secondary for detailed analysis.

MD simulations were performed using GROMACS (Version 2022.4)^38,39^ with the CHARMM36 all-atom force field for proteins^40^ and metformin parameters generated by the CHARMM General Force Field (CGenFF)^41,42^. Each system was solvated in a cubic box containing TIP3P water molecules^36^, maintaining a 10 Å buffer between the solute and box edges, with counterions added to ensure charge neutrality.

Energy minimization was executed using the steepest descent algorithm until forces dropped below 1000 kJ/mol/nm, ensuring complete system relaxation. Equilibration proceeded in two phases: an NVT simulation at 300 K for 100 ps using a velocity-rescaling thermostat^43^, followed by an NPT simulation at 1 atm for 100 ps with the Parrinello-Rahman barostat^44,45^.

Production runs were conducted for 100 ns with a 2 fs time step, employing the Particle Mesh Ewald (PME) method^46^ for long-range electrostatics and a 10 Å cutoff for both electrostatic and van der Waals interactions. Trajectories were recorded every 10 ps for subsequent analysis.

Post-simulation analyses assessed structural stability through root-mean-square deviation (RMSD), residue flexibility via root-mean-square fluctuation (RMSF), and protein compactness through radius of gyration (Rg). Secondary structure analysis was performed using the DSSP (Dictionary of Secondary Structure of Proteins) algorithm to monitor conformational changes in α-helices, β-sheets, and loop regions throughout the simulation trajectories. Hydrogen bond interactions were continuously monitored throughout the simulations.

Binding free energy was calculated using the MM-PBSA approach implemented in the g_mmpbsa tool. The binding free energy (${\Delta G}_{{{\text{Bind}}}}$) was determined using the equation:

2 $$\Delta G_{Binding } = G_{Complex} - \left( {G_{Protein} + G_{Ligand} } \right)$$

where $G_{Complex}$, $G_{Protein}$, and $G_{Ligand}$ are the free energies of the complex, protein, and ligand, respectively^47,48^.

These simulations provided key insights into the dynamic binding behavior of metformin with its targets, revealing structural adaptations that may underlie its moderate binding affinities.

All simulation results were visualized using seaborn, plotly, and matplotlib, generating high-quality interactive graphs for comprehensive interpretation^49–51^.

## Results and discussion

### Formulation and physicochemical characterization of drug-loaded liposomes

#### Entrapment efficiency and drug loading and morphological analysis (TEM)

**Fig. 2:**
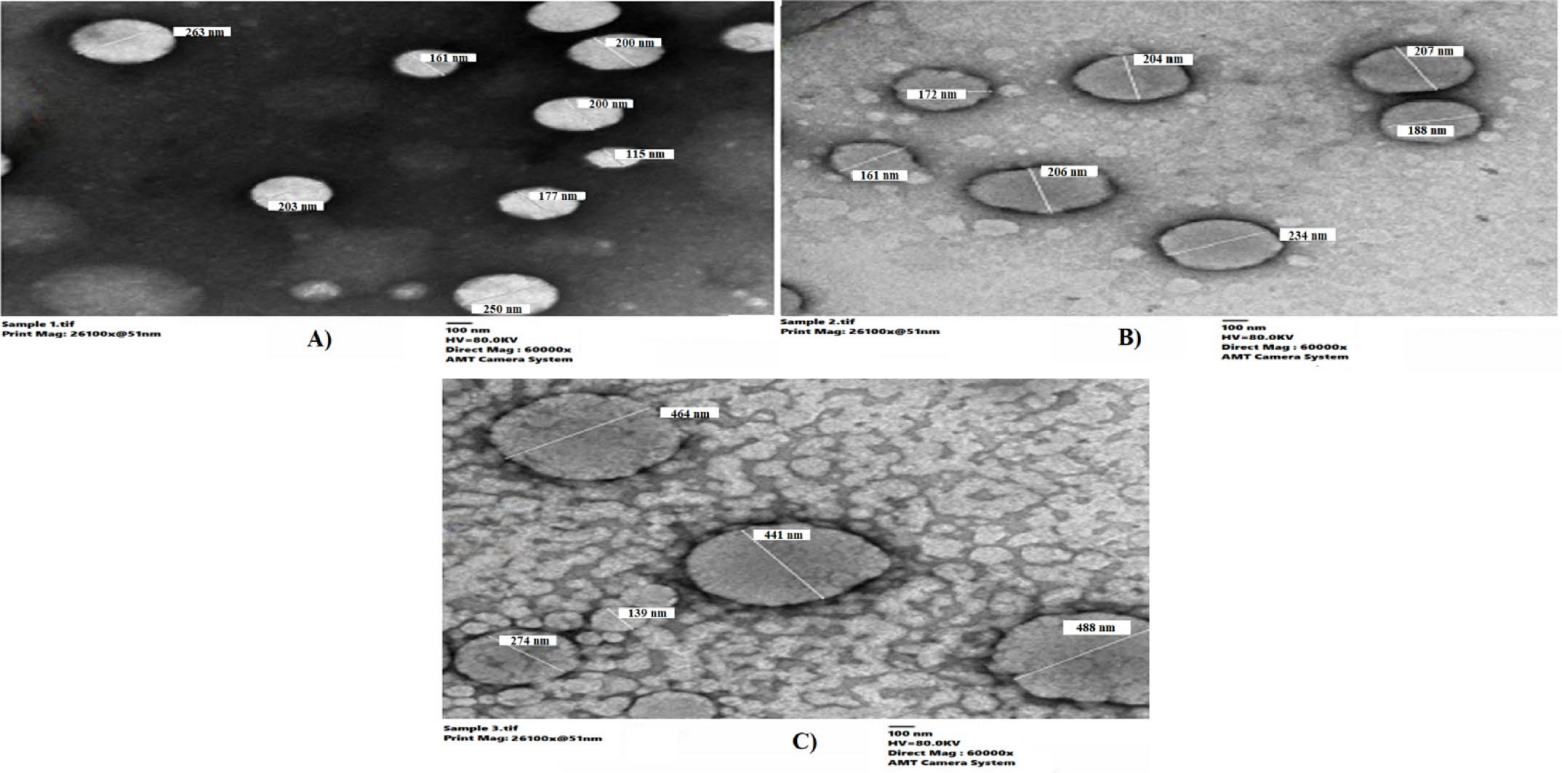
TEM images of (**A**) empty liposomes, (**B**) metformin-loaded liposomes, and (**C**) PEGylated liposomal Metformin.

The entrapment efficiency of all prepared liposomal suspensions exceeded 90% when metformin was premixed with lipid powder prior to ethanol dissolution, demonstrating the effectiveness of this preparation method. Transmission electron microscopy analysis revealed predominantly spherical, well-dispersed vesicles with minimal aggregation across all formulations (Fig. 2A–C). Empty liposomes (Fig. 2A) exhibited a mean diameter of 196.1 ± 47.3 nm, while metformin-loaded vesicles (Fig. 2B) measured 321.2 ± 19.9 nm, and PEGylated metformin liposomes (Fig. 2C) reached 361.6 ± 150.2 nm^52^. These progressive size increases upon drug and PEG incorporation reflect inter-bilayer expansion driven by hydrogen bonding and electrostatic repulsion mechanisms^53–55^. The broad size distribution observed for PEGylated formulations (SD = 150.2 nm) can be attributed to multilamellarity and grid-preparation artifacts, since dehydration during grid prep may induce vesicle fusion or collapse, yielding TEM diameters larger than hydrodynamic values^56^.

#### Dynamic light scattering (DLS) and zeta potential

**Fig. 3:**
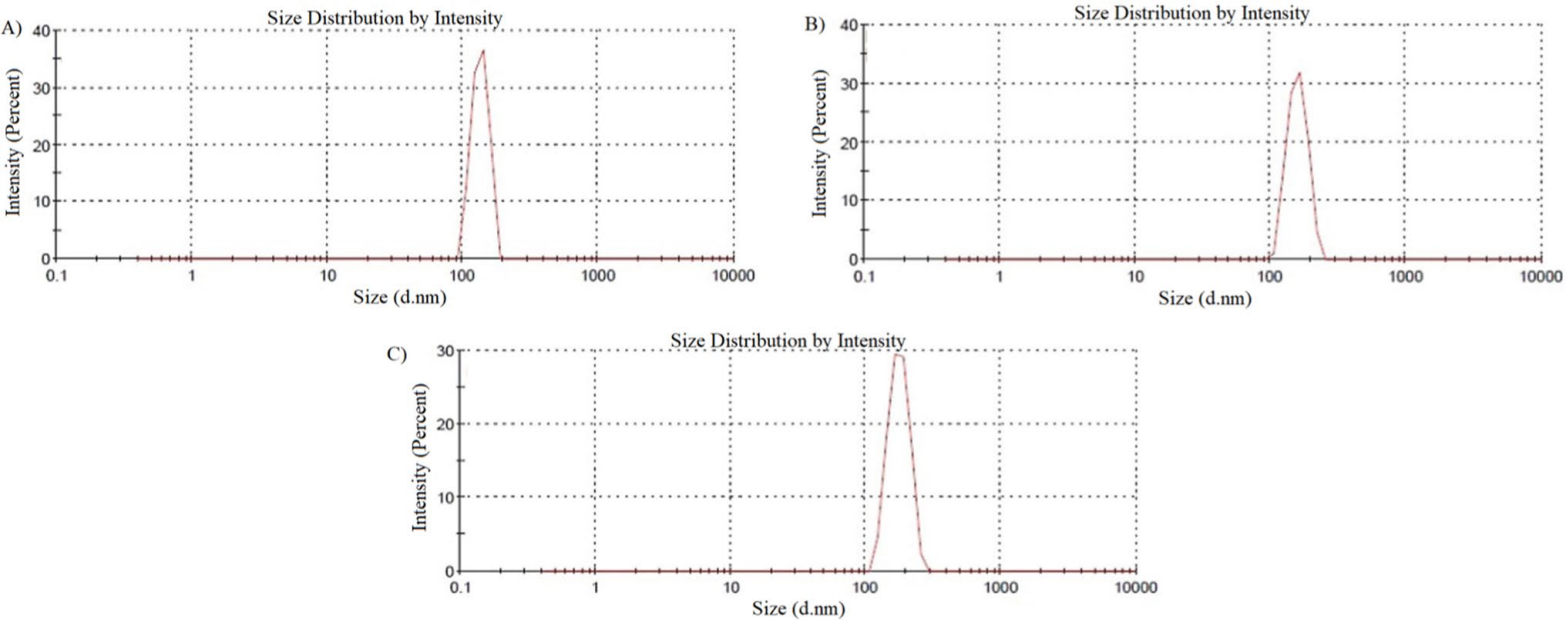
Liposome size distribution measured by dynamic light scattering (DLS) (**A**) empty soy lecithin liposomes, (**B**) Metformin-encapsulated liposomes, and (**C**) PEGylated liposomal Metformin.

Dynamic light scattering (DLS) provided number-weighted hydrodynamic diameters that paralleled our TEM findings while avoiding drying artifacts. Empty soy-lecithin liposomes measured 135.2 ± 18.2 nm (PDI = 0.306), metformin-loaded vesicles 159.5 ± 26.1 nm (PDI = 0.457), and PEGylated metformin liposomes 177.2 ± 30.2 nm (PDI = 0.610) (Fig. 3A–C). A PDI < 0.3 is generally indicative of a homogeneous population, whereas values up to ∼0.5 remain acceptable for proof-of-concept studies; PDIs between 0.5 and 0.7 reflect moderate polydispersity but can be tolerated if batch-to-batch variability is low^52,57^. Indeed, our triplicate preparations yielded coefficients of variation of 3.9%, 4.8%, and 4.3% for empty, metformin-loaded, and PEGylated liposomes, respectively, confirming reproducible size distributions despite moderate heterogeneity. These data demonstrate that drug and PEG incorporation incrementally increase vesicle hydration and steric stabilization without compromising preparation consistency^58^.

Colloidal stability was assessed through zeta potential measurements (Table 1), which revealed moderately negative surface charges across all formulations: − 21.9 ± 3.4 mV for empty vesicles, − 20.6 ± 1.7 mV for metformin-loaded liposomes, and − 22.9 ± 1.5 mV for PEGylated formulations^59^. These values fall within the − 30 to + 30 mV range associated with stable dispersions in physiological media, indicating robust colloidal stability.

**Table 1:** Summarizes the dynamic light scattering (DLS) and zeta potential measurements for the different liposomal formulations.

Sample name	Mean size diameter (nm) ± SD (nm)	PDI average	Mean zeta potential ± SD (mV)
Empty liposomes	135.2 ± 18.23	0.306	− 21.9 ± 3.4
Liposomal metformin	159.5 ± 26.05	0.457	− 20.6 ± 1.7
Pegylated liposomal metformin	177.2 ± 30.23	0.610	− 22.9 ± 1.5

The incorporation of metformin disrupts lipid bilayer packing through multiple mechanisms. Electrostatic repulsion between soy lecithin N(CH₃)₃⁺ groups and metformin NH₃⁺ groups contributes to increased inter-bilayer spacing, while hydrogen bonding facilitates drug insertion into the hydrophobic bilayer core^54,60^. Complementary DSC and FTIR analyses corroborate these structural perturbations, confirming partial entrapment of metformin within the bilayer’s hydrophobic region.

#### Differential scanning calorimetry (DSC)

**Fig. 4:**
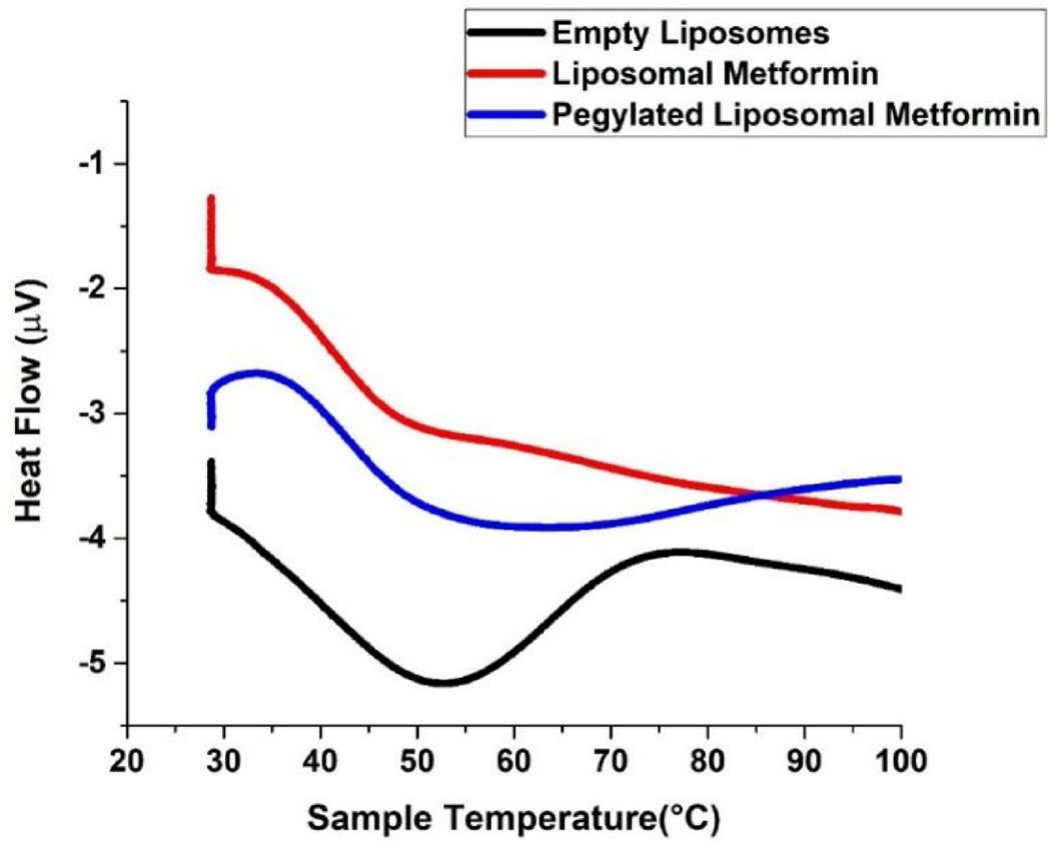
DSC thermograms for liposomes composed of pure soy lecithin, metformin-doped liposomes, and PEGylated liposomal metformin.

Differential scanning calorimetry was employed to analyze the phase transition temperature of the lipid bilayer and elucidate the impact of drug incorporation on liposomal membrane stability. DSC thermograms (Fig. 4) of dehydrated soy lecithin liposomes displayed a main endothermic transition (Tm = 49.2 °C), consistent with previous studies^61–64^. The incorporation of compounds into soy lecithin membranes significantly affects the thermotropic behavior of vesicles. Metformin loading depressed the Tm to 40.1 °C, indicating extensive disruption of acyl-chain packing likely due to drug insertion between polar headgroups and consequent increase in bilayer fluidity^65,66^. This significant decrease implies that metformin, in its amorphous dispersed form within the molten liposomes, interacts extensively with the lipid bilayers, leading to considerable membrane perturbation.

PEGylated metformin liposomes exhibited an intermediate shift (Tm = 47.9 °C), reflecting partial restoration of bilayer order by steric effects of surface-grafted PEG chains. This shift indicates that pegylation exerts a pronounced effect on the acyl chain packing of soy lecithin bilayers, promoting conformational disorder and reducing the cooperative transition of lipid acyl chains^58^. The lowered Tm suggests that the presence of both metformin and PEG favors a more disordered, loosely packed acyl chain arrangement relative to pure soy lecithin control. The single, symmetric DSC peak in both drug-loaded samples confirms homogeneous miscibility of metformin and PEG within the phospholipid matrix^66,67^. This phenomenon may result from insertion of metformin between polar head groups, which destabilizes the orderly gel phase, thereby facilitating transition to a less organized liquid crystalline state.

#### Fourier-transform infrared (FTIR) spectroscopy

**Fig. 5:**
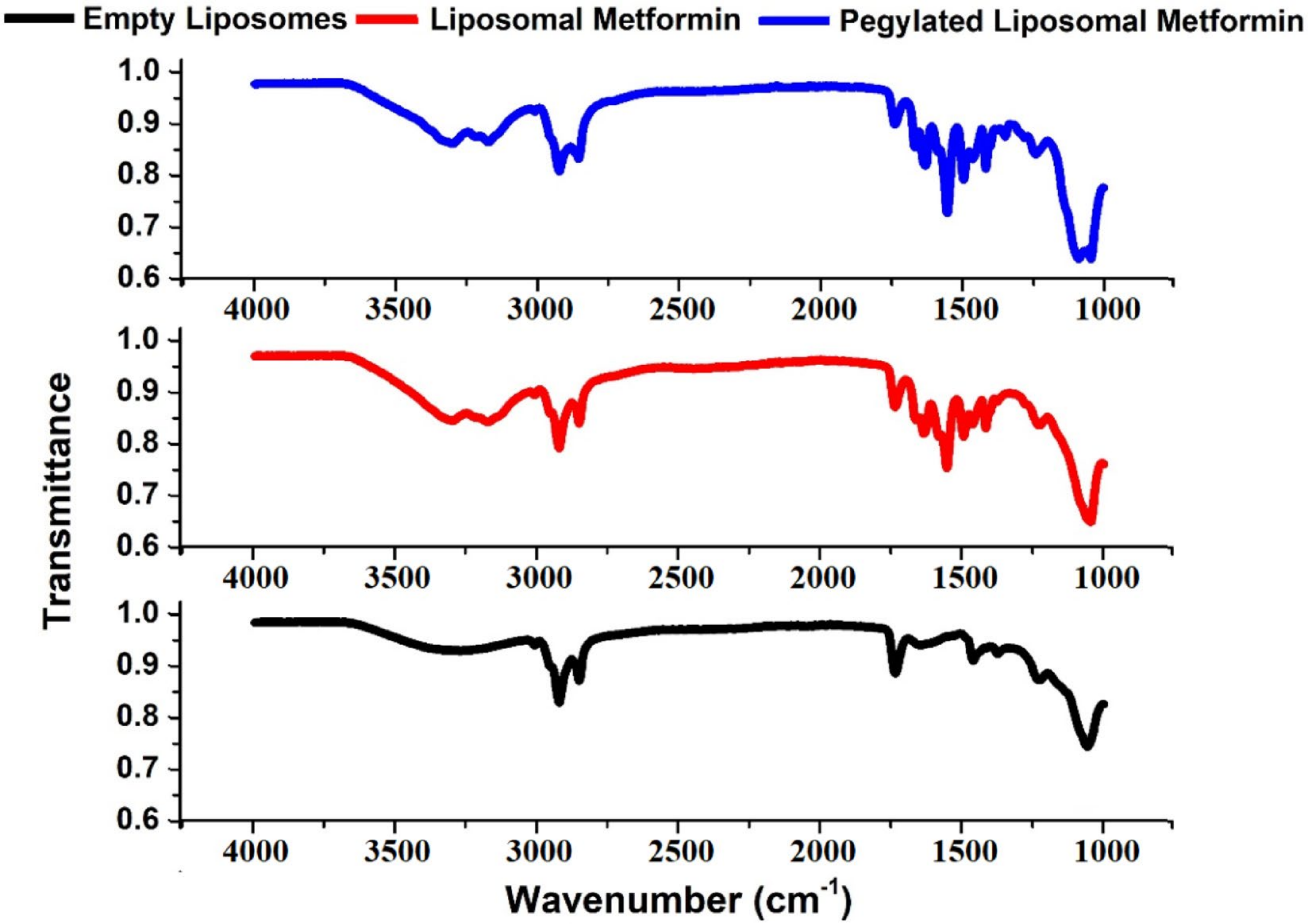
Full FTIR spectra of empty soy lecithin liposomes, liposomes doped with metformin, and PEGylated liposomal metformin.

Fourier-transform infrared spectroscopy was employed to further elucidate structural modifications in the liposomal membrane initially suggested by DSC analysis. FTIR spectra (Fig. 5) of lyophilized liposomes were acquired over 4000–400 cm⁻^1^ to probe bilayer structural changes upon drug and PEG incorporation (Table 2). In empty vesicles, the symmetric CH₂ stretch appeared at 2852.7 cm⁻^1^. When metformin was encapsulated, a notable shift was observed with the peak moving to 2846.08 cm⁻^1^ in metformin-loaded samples. This downshift from 2852.7 to 2846.08 cm⁻^1^ indicates an increase in gauche conformers within the acyl chains, suggesting that metformin disrupts phospholipid chain packing, which decreases membrane order and is consistent with the observed depression in phase transition temperature from DSC analysis^54^. PEGylation reversed this shift back to 2852.7 cm⁻^1^, consistent with increased chain disorder imparted by surface-anchored PEG.

**Table 2:** Chemical shifts observed for metformin and PEGylated metformin after incorporation into soy lecithin liposomes.

Peak assignment	Wavenumber (cm^−1^)	Wavenumber (cm^−1^)		
		Empty	Liposomal metformin	Pegylated liposomal metformin
Symmetric stretching vibration of CH_2_ in acyl chain	(2800–2855)	2852.7	2846.08	2852.7
Antisymmetric stretching vibration of CH_2_ in acyl chain	(2916–2921)	2920.08	2920. 08	2920.08
Carbonyl stretching vibration C = O	(1730–1740)	1735.28	1741.08	1735.28
Antisymmetric PO_2_^−^ stretching vibrations	(1215–1260)	1235.38	1223.05	1235.38

Significant changes were noted in the carbonyl (C = O) stretching region, which is sensitive to the local environment around the glycerol backbone. In liposomes loaded solely with metformin, the C = O stretching frequency increased from 1735.28 cm⁻^1^ in empty liposomes to 1741.08 cm⁻^1^, suggesting altered local polarity and strengthened hydrogen bonding around ester linkages^68^. This upward shift indicates that metformin presence alters local polarity around ester groups, modulating hydrogen-bonding interactions without necessarily forming new bonds.

The PO₂⁻ antisymmetric stretching band, a marker for the lipid head group region, shifted from 1235.38 cm⁻^1^ in empty liposomes to 1223.05 cm⁻^1^ after metformin incorporation, confirming enhanced drug-headgroup interactions at the polar/apolar interface. This shift clearly indicates that hydrogen bonding between metformin and lipid head groups is either established or strengthened, contributing to stabilization of liposomal structure. PEGylated samples returned to 1235.38 cm⁻^1^, indicating that PEG sterically shields headgroup hydrogen bonds^53^. These FTIR findings corroborate DSC results by demonstrating that metformin and PEG differentially modulate bilayer order: metformin promotes headgroup interaction and tighter packing, whereas PEG enhances surface hydration and chain mobility, together supporting a finely tunable liposomal architecture for controlled release.

The incorporation of metformin disrupts lipid bilayer packing through multiple mechanisms. Electrostatic repulsion between soy lecithin N⁺(CH₃)₃ groups and metformin NH₃⁺ groups contributes to increased inter-bilayer spacing, while hydrogen bonding facilitates drug insertion into the hydrophobic bilayer core. Although metformin is primarily hydrophilic, partial entrapment within the hydrophobic region was confirmed through complementary DSC and FTIR analyses, providing compelling evidence that metformin incorporation induces distinct structural modifications in liposomal membranes, contributing to improved membrane stability and potentially enhanced drug delivery efficacy.

### Biological activity and mechanistic evaluation of metformin formulations

#### In vitro assessment of liposomal metformin’s cytotoxic profile

**Fig. 6:**
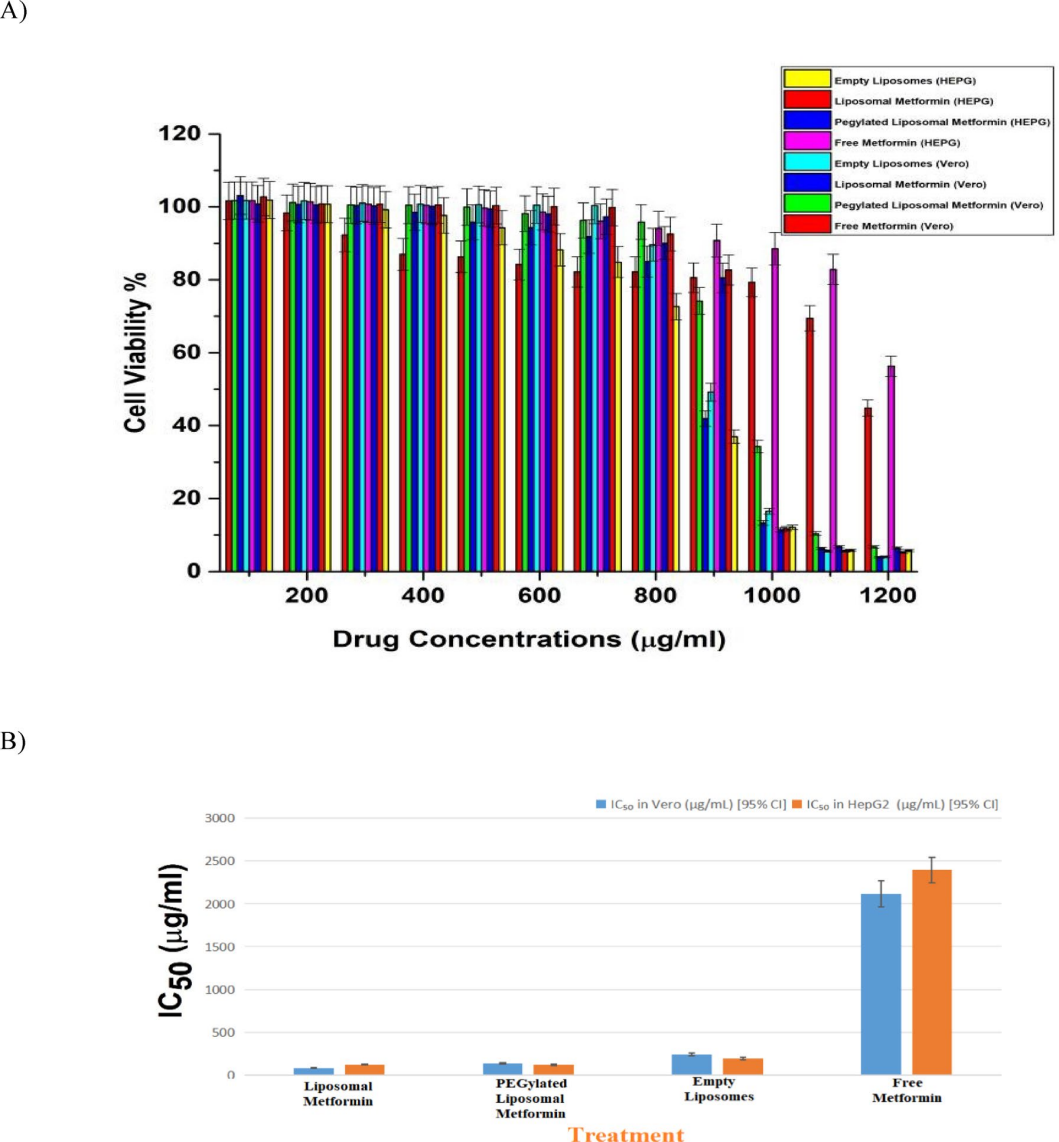
In vitro cytotoxicity evaluation of metformin formulations against normal (Vero) and liver carcinoma (HepG2) cell lines. (**A**) Dose–response curves showing cell viability after 48 h incubation with drug concentrations from 100 to 1200 µg/mL, assessed via MTT assay.Comparative IC₅₀ values derived from dose–response curves demonstrating enhanced potency of liposomal formulations. Data represent mean ± standard error from three independent experiments.

In 48-h MTT assays, both PEGylated and non-PEGylated liposomal metformin formulations exhibited significantly greater cytotoxicity against HepG2 hepatocellular carcinoma cells compared to free metformin. At the highest tested concentration of 1000 µg/mL, PEGylated liposomal metformin reduced HepG2 cell viability to 6.46%, while the non-PEGylated formulation reduced it further to 5.30% (Fig. 6). Post-hoc comparisons confirmed the superior efficacy of both liposomal formulations over free metformin (*p* ≤ 0.0001). A two-way ANOVA conducted across both cell lines demonstrated a highly significant main effect of drug concentration (F(1,80) = 199.32, *p* < 1.99e − 23), a significant interaction between formulation and concentration (F(3,80) = 8.01, *p* < 9.89e − 05), and a significant main effect of formulation type (F(3,80) = 4.08, *p* = 0.0095), indicating that cytotoxicity is concentration-dependent and formulation-specific, with more pronounced differences observed at higher doses.

In non-cancerous Vero kidney epithelial cells, IC₅₀ values were 137.13 µg/mL for PEGylated liposomal metformin and 82.46 µg/mL for the non-PEGylated variant, while free metformin demonstrated markedly lower toxicity with an IC₅₀ of 2113.86 µg/mL (Table 3, Fig. 6B). Although free metformin displayed relatively high IC₅₀ values in HepG2 cells (2392.81 µg/mL), these values suggest that it possesses some intrinsic cytotoxic potential. However, this effect occurs only at suprapharmacological concentrations, which are clinically unachievable and thus render the free form therapeutically nonviable for oncology applications.

**Table 3:** IC₅₀ values and calculated Selectivity Index (SI) for metformin formulations in HepG2 and Vero cell lines.

Formulation	Mean IC₅₀ in Vero (µg/mL) [95% CI]	Mean IC₅₀ in HepG2 (µg/mL) [95% CI]	Selectivity Index (SI)
Liposomal metformin	82.46	121.27	0.68
PEGylated liposomal metformin	137.13	118.76	**1.1546**
Empty liposomes	240.72	189.49	**1.27**
Free metformin	2113.86	2392.81	0.88

Data represents mean values with 95% confidence intervals from three independent experiments. SI values below 1.0 indicate preferential toxicity to normal cells over cancer cells.

Significant values are in [bold].

The selectivity index (SI), calculated as the IC₅₀ in Vero cells divided by the IC₅₀ in HepG2 cells, further supports this interpretation. PEGylated liposomal metformin exhibited an SI of 1.15, while the non-PEGylated formulation had an SI of 0.68—values indicative of moderate selectivity. Free metformin showed an SI of 0.88, which, while higher than the non-PEGylated formulation, remains biologically insignificant due to the requirement of excessively high dosing for efficacy.

At 1000 µg/mL, both liposomal formulations significantly reduced Vero cell viability to 6.75% and 3.95%, respectively (*p* < 0.0025 and *p* < 0.0001 vs. free metformin), raising concerns about off-target toxicity at supratherapeutic levels. Empty liposomes alone also induced substantial cytotoxicity, decreasing viability to 5.73% in HepG2 and 4.08% in Vero cells, likely due to lipid membrane destabilization rather than a drug-specific mechanism.

Thus, despite free metformin demonstrating cytotoxicity at high concentrations, its therapeutic window is too narrow to be clinically useful for cancer treatment. Both liposomal formulations reduced HepG2 viability relative to free metformin; PEGylated vs non-PEGylated IC₅₀ values were similar (118.76 vs 121.27 µg/mL), and selectivity over Vero cells was modest (SI ≈ 1.15). Importantly, empty liposomes alone lowered viability in both HepG2 and Vero cells at high concentrations, indicating a vehicle contribution to cytotoxicity. Because drug-release and uptake kinetics were not measured, mechanistic attributions remain speculative and have been removed; targeted studies are needed to distinguish lipid versus drug effects.

#### Quantitative analysis of comet assay

**Fig. 7:**
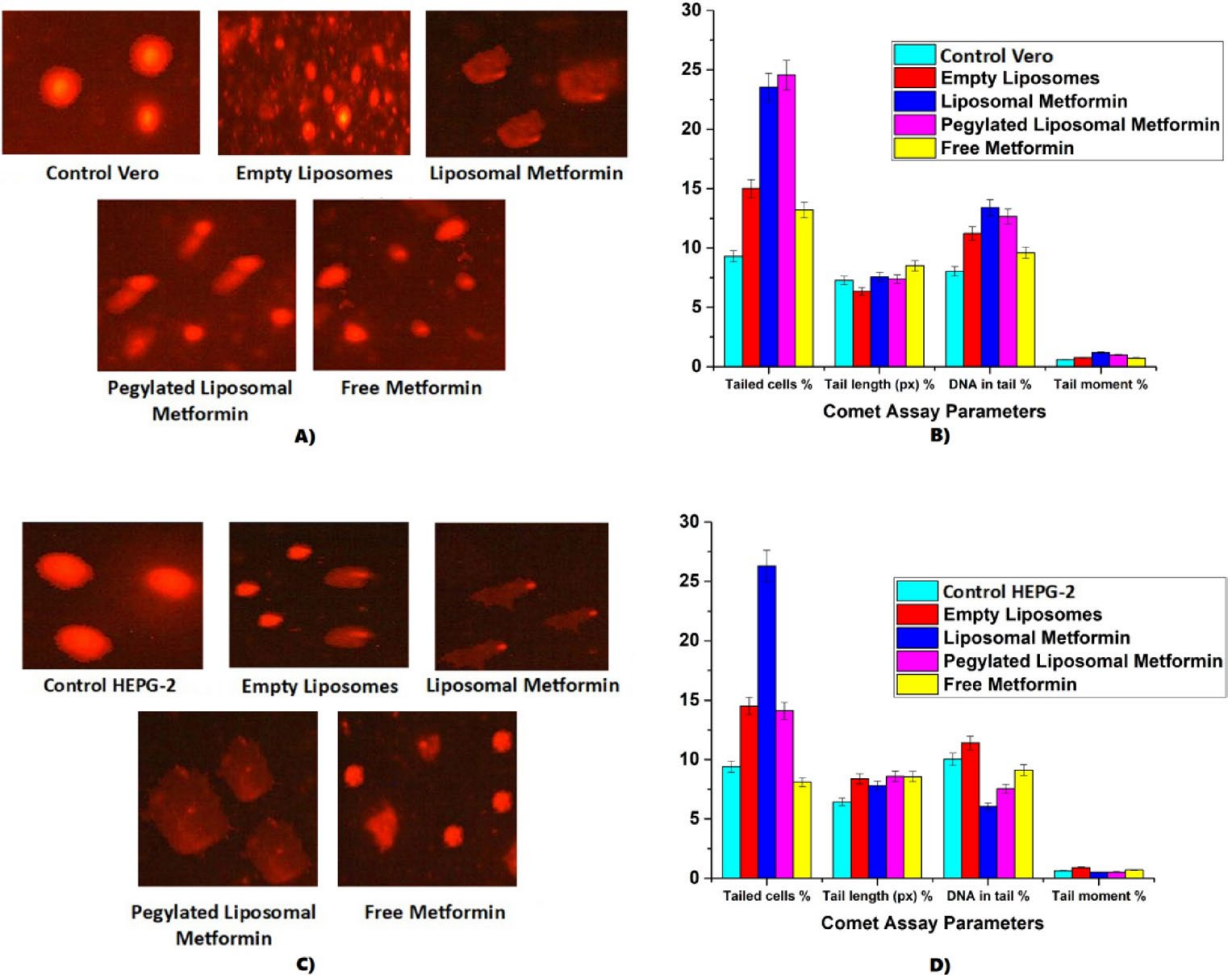
Comet assay analysis of DNA damage in normal Vero and liver carcinoma HEPG-2 cells following treatment with Free Metformin, Empty Liposomes, Liposomal Metformin, and Pegylated Liposomal Metformin. (**A**) Representative fluorescence microscopy images of Vero cells, showing comet tails indicative of DNA damage. (**B**) Bar graph of quantitative comet assay parameters for Vero cells, including percentage of tailed cells, tail length (pixels), % DNA in tail, and tail moment. (**C**) Representative fluorescence microscopy images of HEPG-2 cells, highlighting DNA fragmentation via comet tails. (**D**) Bar graph of quantitative comet assay parameters for HEPG-2 cells, detailing the same metrics. Data are expressed as mean ± standard error from three independent experiments. Statistical significance was assessed using one-way ANOVA with Tukey’s post-hoc test (**p < 0.05, **p < 0.01, ***p < 0.001 vs. control). Both liposomal formulations exhibited the highest percentage of tailed cells across both cell lines, with differential effects on DNA damage intensity parameters between normal and cancer cells.

In HEPG-2 cells, Liposomal Metformin significantly increased the percentage of tailed cells to 26.3 ± 1.41% (95% CI: 23.5–29.1) compared to the control (9.4 ± 0.14%, 95% CI: 9.1–9.7, *p* < 0.0001). This increase in DNA damage is clearly illustrated in Fig. 7 (Panel C), where representative fluorescence microscopy images show numerous HEPG-2 cells with pronounced comet tails, indicating substantial DNA fragmentation. However, the extent of DNA damage per cell, measured as % DNA in tail, was significantly lower in the Liposomal Metformin group (6.04 ± 0.13%, 95% CI: 5.78–6.30, *p* = 0.0056) compared to the control (10.04 ± 0.47%, 95% CI: 9.12–10.96). Similarly, the Pegylated Liposomal Metformin group showed significantly lower % DNA in tail (7.54 ± 0.20%, 95% CI: 7.15–7.93, *p* < 0.0001) compared to control. Tail moment values were also significantly reduced in the Liposomal Metformin group (0.52 ± 0.12, 95% CI: 0.28–0.76, *p* = 0.0326) and Pegylated Liposomal Metformin group (0.53 ± 0.03, 95% CI: 0.47–0.59, *p* = 0.0308) relative to the control (0.64 ± 0.06, 95% CI: 0.53–0.75). Pegylated Liposomal Metformin significantly increased the percentage of tailed cells to 14.1 ± 0.36% (95% CI: 13.4–14.8, *p* < 0.0001), while Free Metformin showed a significant but smaller effect (8.1 ± 0.36%, 95% CI: 7.4–8.8, *p* = 0.0036) with % DNA in tail at 9.13 ± 1.5% (95% CI: 6.20–12.06, *p* = 0.0225) and tail moment at 0.70 ± 0.03 (95% CI: 0.64–0.76, *p* = 0.1822, not significant). Empty liposomes also significantly elevated tailed cells to 14.5 ± 0.5% (95% CI: 13.5–15.5, *p* = 0.0002), with % DNA in tail at 11.39 ± 1.37% (95% CI: 8.71–14.07, *p* = 0.0001) and tail moment at 0.90 ± 0.01 (95% CI: 0.88–0.92, *p* = 0.5709, not significant), above control levels. These findings indicate that while Liposomal Metformin markedly increases the proportion of damaged HEPG-2 cells, the severity of DNA fragmentation per cell remains paradoxically lower than in untreated controls, suggesting a complex mechanism where cellular population heterogeneity may influence damage distribution patterns.

In normal Vero cells, both Liposomal Metformin and Pegylated Liposomal Metformin induced substantial DNA damage, as evidenced by quantitative data and fluorescence images. Figure 7 (Panel A) provides representative fluorescence microscopy images of Vero cells, where comet tails are visible, indicating DNA damage across the treatment groups. The Liposomal Metformin group exhibited a highly significant rise in tailed cells to 23.53 ± 0.5% (95% CI: 22.5–24.5, *p* < 0.0001) compared to the control (9.3 ± 0.3%, 95% CI: 8.7–9.9), with elongated comet tails visible in the images. The % DNA in tail reached 13.4 ± 0.4% (95% CI: 12.6–14.2, *p* = 0.0918, approaching significance) and tail moment 1.2 ± 0.17 (95% CI: 0.87–1.53, *p* = 0.0274), with the tail moment being significantly higher than the control’s 0.59 ± 0.18 (95% CI: 0.55–0.63). Pegylated Liposomal Metformin showed comparable genotoxicity, with tailed cells at 24.56 ± 0.5% (95% CI: 23.6–25.5, *p* < 0.0001), % DNA in tail at 12.65 ± 0.65% (95% CI: 11.4–13.9, *p* = 0.0176), and tail moment at 1.0 ± 0.06 (95% CI: 0.88–1.12, *p* = 0.0542, approaching significance), exceeding control values. Free Metformin caused a significant increase in tailed cells to 13.2 ± 0.3% (95% CI: 12.6–13.8, *p* < 0.0001), with % DNA in tail at 9.6 ± 0.27% (95% CI: 9.1–10.1, *p* = 0.3127, not significant) and tail moment at 0.72 ± 0.22 (95% CI: 0.29–1.15, *p* = 0.0740, not significant), above control but below nano-formulations. Empty liposomes also significantly elevated DNA damage, with tailed cells at 15 ± 0.5% (95% CI: 14.0–16.0, *p* < 0.0001), % DNA in tail at 11.22 ± 0.5% (95% CI: 10.2–12.2, *p* = 0.0032), and tail moment at 0.76 ± 0.066 (95% CI: 0.63–0.89, *p* = 0.0740, not significant). These results confirm that nano-formulations significantly enhance DNA fragmentation in Vero cells, with the most pronounced effects observed in percentage of tailed cells and selective effects on DNA damage intensity parameters, consistent with their enhanced cellular uptake efficiency compared to free drug formulations.

#### Apoptosis induction in HEPG-2 and normal (vero) cells following treatment with different metformin formulations

To elucidate the cytotoxic mechanisms of various Metformin formulations, apoptosis and necrosis were assessed in HEPG-2 liver carcinoma cells and normal Vero cells using Annexin V-FITC/PI staining and flow cytometry after 48 h of treatment at their respective IC₅₀ concentrations.

In HEPG-2 cells, PEGylated liposomal Metformin induced the highest level of apoptosis, with total apoptosis (early + late) at 20.67% (13.08% early, 7.59% late), followed by free Metformin at 15.8% (4.33% early, 11.47% late), empty liposomes at 11.17% (9.29% early, 1.88% late), and liposomal Metformin at 9.57% (3.51% early, 6.06% late). The untreated control showed only 0.61% apoptosis (0.37% early, 0.24% late). Necrosis was also elevated with PEGylated liposomal Metformin at 4.77%, compared to 2.58% for free Metformin, 3.86% for empty liposomes, 2.34% for liposomal Metformin, and 1.94% for the control.

In Vero cells, PEGylated liposomal Metformin induced 4.69% apoptosis (2.92% early, 1.77% late) and 4.95% necrosis, while free Metformin induced 4.47% apoptosis (2.69% early, 1.78% late) and 3.32% necrosis, empty liposomes induced 2.76% apoptosis (1.88% early, 0.9% late) and 3.1% necrosis, and liposomal Metformin induced 2.15% apoptosis (1.33% early, 0.82% late) and 2.77% necrosis. The control showed 0.52% apoptosis (0.33% early, 0.19% late) and 2.03% necrosis.

**Fig. 8:**
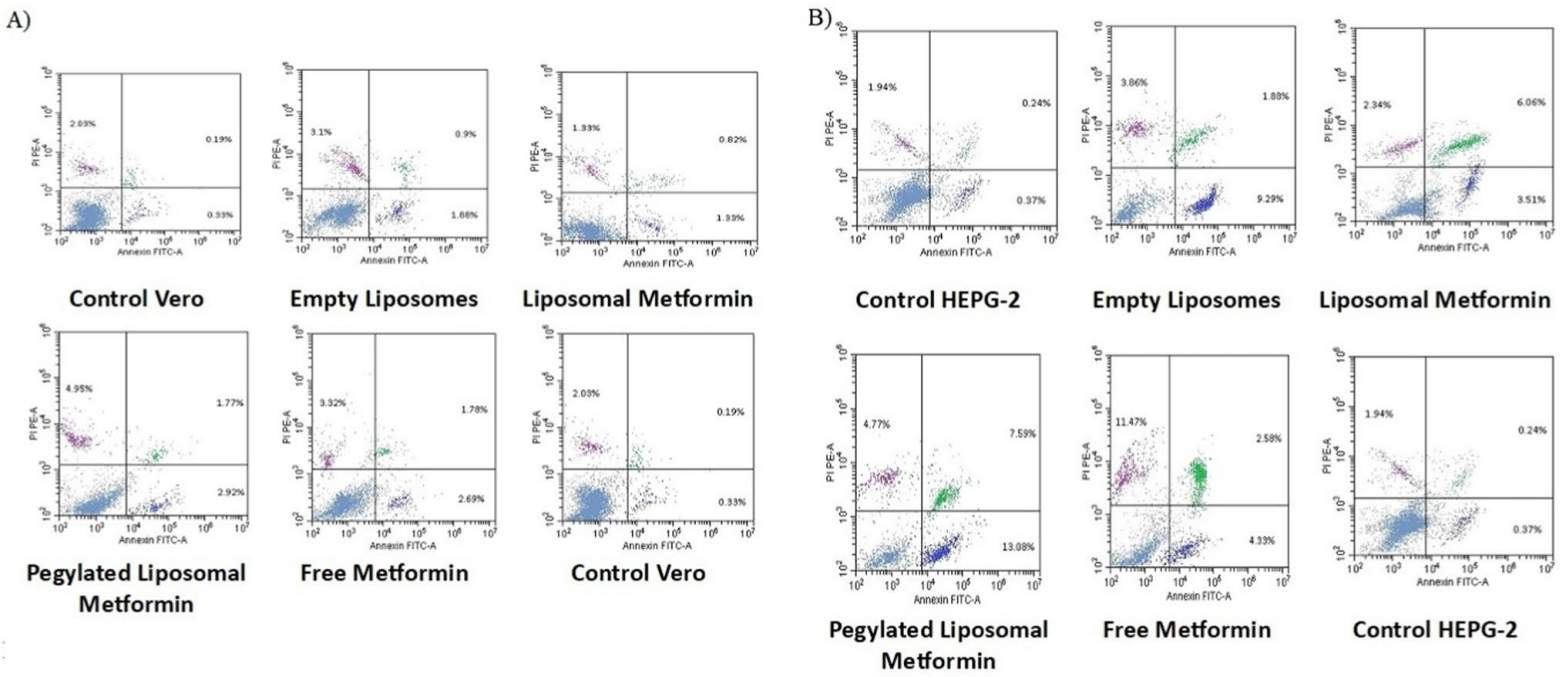
Flow cytometric analysis of apoptosis using Annexin V-FITC/PI staining in (**A**) HEPG-2 and (**B**) Vero cells after 48 h of treatment with free metformin, empty liposomes, liposomal metformin, and PEGylated liposomal metformin at their respective IC₅₀ concentrations.

Statistical analysis using one-way ANOVA revealed highly significant treatment effects for both apoptosis (HEPG-2: F₄,₁₀ = 170.57, *p* = 3.728 × 10⁻⁹; Vero: F₄,₁₀ = 148.81, *p* = 7.287 × 10⁻⁹) and necrosis (HEPG-2: F₄,₁₀ = 1007.7, *p* = 5.558 × 10⁻^13^; Vero: F₄,₁₀ = 347.62, *p* = 1.107 × 10⁻^1^⁰). Tukey’s post-hoc analysis confirmed that all treatments significantly increased both apoptosis and necrosis compared to controls in both cell lines (all *p* < 0.05) (Fig. 8).

#### Cell cycle modulation in cancerous and normal cells induced by metformin formulations

In addition to apoptosis, one significant strategy for controlling the growth of cancer cells is to stop the advancement of the cell cycle. Chemopreventive drugs can also induce cell cycle arrest, which may be a useful treatment for uncontrolled cell proliferation and survival in tumor cells. Thus, as potential therapies for reducing the growth of cancer cells, a large variety of naturally occurring substances that target cell cycle regulatory components have been studied^69^.

**Fig. 9:**
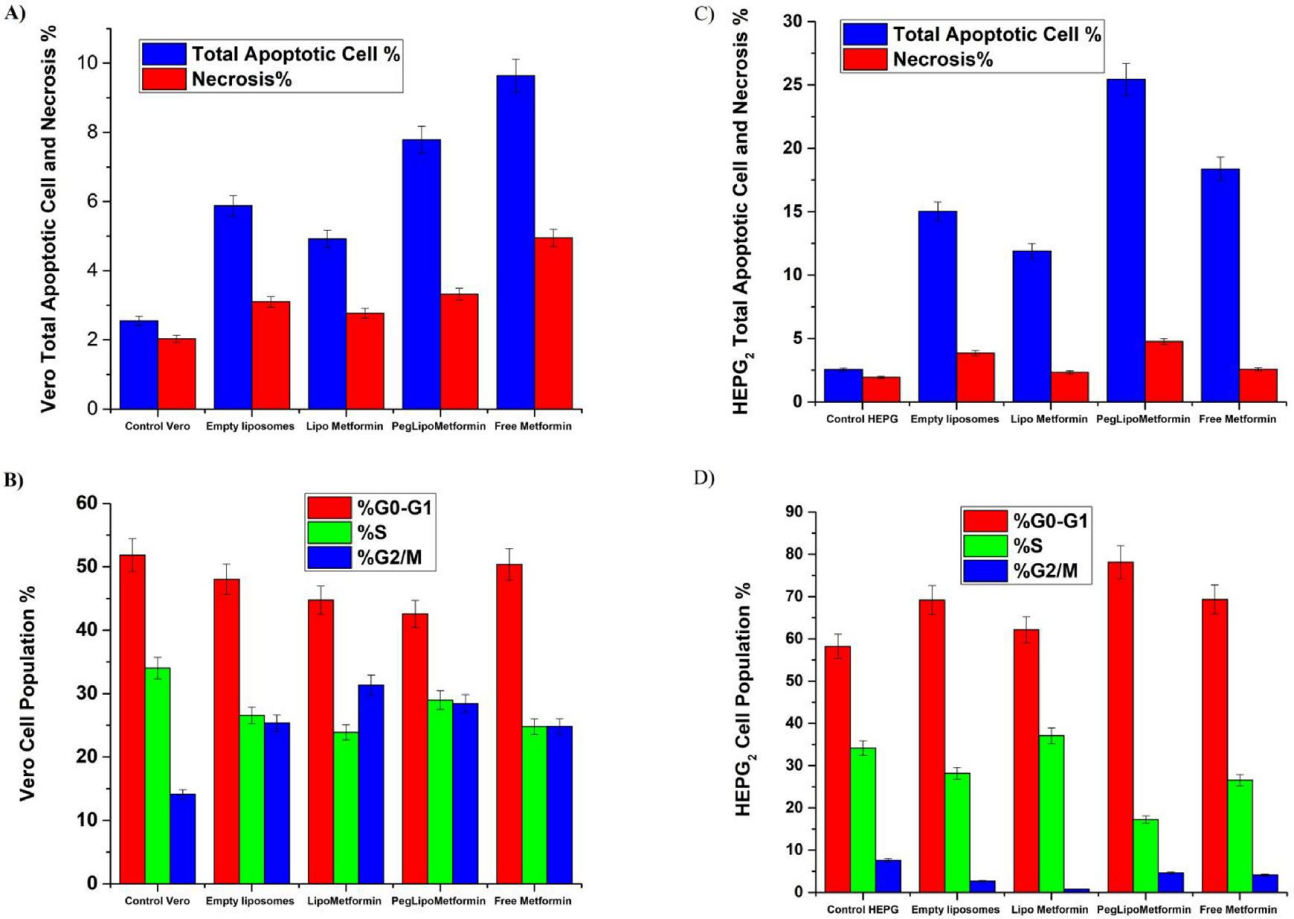
Effects of various metformin formulations on Vero and HEPG-2 cells after 48 h of treatment. (**A**) Apoptosis and necrosis in Vero cells (blue: apoptosis, red: necrosis), (**B**) cell cycle distribution in Vero cells (red: G0-G1, green: S, blue: G2/M), (**C**) apoptosis and necrosis in HEPG-2 cells (blue: apoptosis, red: necrosis), (**D**) cell cycle distribution in HEPG-2 cells (red: G0-G1, green: S, blue: G2/M). Data are presented as mean ± SD.

Flow cytometric analysis was employed to examine cell cycle phase distribution in HEPG-2 and Vero cells following 48 h of treatment with Metformin formulations at IC₅₀ concentrations, with results presented in Fig. 9. In HEPG-2 cells, PEGylated liposomal Metformin induced a robust G0/G1 arrest, with 78.12% of cells in G0-G1 and 17.26% in S phase, compared to the control’s 58.21% and 34.18%, respectively. Free Metformin also promoted G1 arrest, albeit less potently, at 69.33% G0-G1 and 26.56% S, whereas liposomal Metformin increased the S phase to 37.08% (vs. 34.18% in control), and empty liposomes slightly elevated G1 to 62.15%.

Statistical analysis revealed significant treatment effects on cell cycle distribution in HEPG-2 cells across all phases: G0/G1 (F₄,₁₀ = 8.57, *p* = 0.003), S phase (F₄,₁₀ = 28.32, *p* = 1.966 × 10⁻^5^), and G2/M phase (F₄,₁₀ = 1884.9, *p* = 2.444 × 10⁻^14^). Tukey’s post-hoc analysis showed that PEGylated liposomal Metformin (s3) produced the most pronounced effects, with highly significant differences from control in S phase (*p* = 0.0001) and from other treatments in G2/M phase (all *p* < 0.0001).

Conversely, in Vero cells, the overall effect on G0/G1 phase distribution was not statistically significant (F₄,₁₀ = 1.83, *p* = 0.200), indicating that normal cells do not exhibit the same G1 arrest response observed in cancer cells. However, significant treatment effects were observed for S phase (F₄,₁₀ = 7.93, *p* = 0.004) and G2/M phase (F₄,₁₀ = 12.86, *p* = 0.0006). All formulations shifted the cell cycle toward G2/M accumulation, with libosomal Metformin exhibiting the highest G2/M fraction at 31.36%, compared to the control’s 14.15%, with PEGylated liposomal Metformin, free Metformin, and empty liposomes showing 24.83%, 28.44%, and 25.37%, respectively. Tukey’s post-hoc analysis confirmed significant increases in S phase for multiple treatments compared to control: free Metformin (*p* = 0.029), empty liposomes (*p* = 0.004), and PEGylated liposomal Metformin (*p* = 0.008).

These findings demonstrate differential cell cycle responses between cancerous and normal cells, with HEPG-2 cells exhibiting significant G1 arrest particularly with PEGylated formulations, while Vero cells show G2/M accumulation without significant G1 changes. Flow cytometry revealed distinct death modes: PEGylated liposomal metformin produced the highest apoptotic fraction in HepG2 cells (total ~ 21%), whereas free metformin treatment showed a larger necrotic component. Because comparable apoptosis increases were not observed in Vero cells, these differences may reflect formulation-specific stress responses rather than improved ‘therapeutic selectivity’; additional validation with apoptotic and necrotic controls is required.

### In silico results

#### In silico pharmacokinetic predictions

In silico ADME/T analysis revealed that metformin possesses a distinctly favorable pharmacokinetic and safety profile for hepatocellular carcinoma (HCC) compared to the first-line tyrosine-kinase inhibitors sorafenib and lenvatinib (Table 4). Metformin’s low lipophilicity (log P = 0.06) combined with a moderate topological polar surface area (TPSA = 91.5 Å^2^) predicts excellent aqueous solubility and sufficient membrane permeability for oral dosing and efficient hepatic uptake^70,71^. Predicted OATP1B1/1B3 transport probabilities (0.21/0.82) indicate active hepatocyte entry without overwhelming non-specific tissue retention, while negligible BSEP inhibition (1.6 × 10⁻⁷) suggests minimal disruption of bile acid homeostasis—a key consideration in cirrhotic HCC patients. By contrast, sorafenib and lenvatinib display high lipophilicity (log P > 2.9) and near-maximal BSEP inhibition (> 0.99), consistent with reported hepatotoxicity in clinical use^72^.

**Table 4:** In silico PK parameters tailored to HCC pharmacology.

Parameter	Metformin	Sorafenib	Lenvatinib
Molecular Weight (Da)	129.1	464.09	426.11
log P (lipophilicity)	0.06	4.68	2.89
TPSA (Å^2^)	91.49	92.35	115.57
OATP1B1 uptake prob	0.21	0.99	0.998
OATP1B3 uptake prob	0.82	0.99995	0.99996
BSEP inhibition prob	1.6 × 10⁻⁷	0.9999998	0.994
P-gp substrate prob	0.9994	0.0047	0.2131
P-gp inhibition prob	0.0001	0.9678	0.0543
BCRP substrate prob	1.8 × 10⁻^5^	0.000012	0.00699
CYP3A4 inhibition prob	1.6 × 10⁻^11^	0.0602	0.3924
hERG blockade prob	0.21	0.63	0.64
BBB permeation prob	0.53	0.00264	0.000223

Metformin’s rapid plasma clearance (6.30 mL/min/kg) and low volume of distribution (log VDss =  − 0.24) further mitigate the risk of systemic accumulation, whereas the kinase inhibitors’ slower clearance and extensive tissue distribution may prolong off-target exposure. Critically, metformin’s hERG blockade probability (0.21) is substantially lower than that of sorafenib (0.63) and lenvatinib (0.64), forecasting a markedly reduced risk of QT prolongation and cardiotoxicity^71^. Additionally, metformin exhibits negligible CYP3A4 inhibition (1.6 × 10⁻^11^), minimizing potential drug–drug interactions in the polypharmacy context of advanced HCC management, unlike sorafenib (0.06) and lenvatinib (0.39).

Taken together, these data support metformin’s repurposing for HCC therapy, combining efficient hepatocyte targeting with superior safety margins. We recommend progression to HCC-specific preclinical validation—such as patient-derived tumor spheroids and orthotopic murine models—to confirm intra-tumoral drug accumulation, antitumor efficacy, and tolerability. Such studies will enable optimization of dosing strategies that maximize on-target activity while preserving residual liver function and minimizing cardiovascular risk.

#### Molecular interactions and docking results

**Fig. 10:**
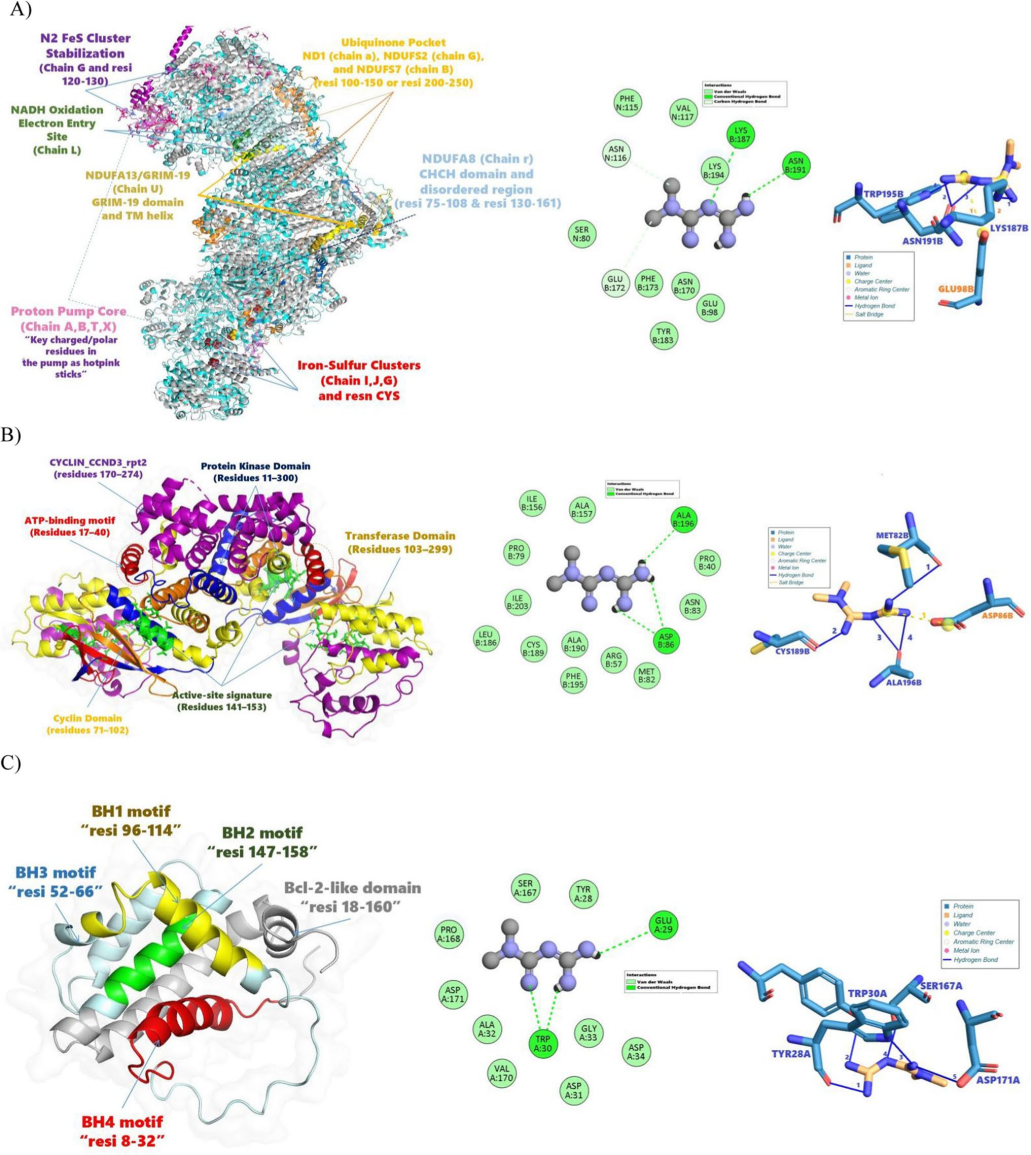
Metformin’s 3D and 2D Interaction Profiles with Target Proteins and Structural and Functional Annotation. (**A**) Mitochondrial Complex I (PDB: 5XTD). (**B**) CDK4/cyclin D3 (PDB: 3G33). (**C**) Bcl-2 (PDB: 1G5M).

Metformin docking into human mitochondrial Complex I (5XTD) reveals critical interactions with structurally and functionally defined domains, as illustrated in Fig. 10A. Key findings highlight binding at the ubiquinone pocket (formed by ND1 (chain a), NDUFS2 (chain G), and NDUFS7 (chain B)), where metformin occupies a hydrophobic cavity flanked by residues LYS187B , GLU98B , and TRP195B (distance ~ 3.5 Å from LYS187B’s ε-NH2 group). This positioning suggests interference with ubiquinone binding, potentially disrupting electron transfer.

Metformin also interacts with the proton pump core (chains A, B, T, X), where charged residues (e.g., SER80 , GLU172B ) form hydrogen bonds with metformin’s nitrogen atoms (angle ~ 120°), stabilizing the inhibitor within the membrane arm. Notably, metformin’s proximity to the N2 FeS cluster stabilization region (chain G, residues 120–130) implies perturbation of iron-sulfur coordination, risking redox dysfunction. Structural flexibility in the NDUFA8 disordered region (residues 130–161) may facilitate metformin access to buried pockets, while the GRIM-19 domain (chain U) remains unaffected, preserving Complex I asSDbly. These interactions align with metformin’s proposed mechanism of inhibiting oxidative phosphorylation by blocking ubiquinone reduction and proton translocation.

The docking analysis of metformin with the CDK4 cyclin D3 complex (PDB: 3G33) reveals critical interactions within the protein kinase domain (residues 11–300), as depicted in Fig. 10B. Metformin binds near the ATP-binding motif (residues 17–40) and active-site signature (residues 141–153), forming hydrogen bonds with key residues such as ASP86, MET82, and CYS189 (distance: ~ 2.5–3.2 Å). These interactions stabilize the ligand within the hydrophobic pocket, disrupting ATP binding and potentially inhibiting kinase activity. Notably, metformin’s aromatic ring stacks with PHE195 via π-π interactions (distance: ~ 4.1 Å), enhancing binding affinity. The angle between metformin’s nitrogen atoms and backbone carbonyls of ALA196 and ILE156 (~ 120°) optimizes electrostatic complementarity. Additionally, van der Waals contacts with LEU186 and PRO40 further anchor the ligand. This binding mode suggests metformin acts as an ATP-competitive inhibitor, blocking substrate access to the catalytic site. Structural flexibility in the transferase domain (residues 103–299) may also be perturbed, altering the overall conformation to inhibit cyclin-dependent kinase 4 (CDK4) activity. The short binding distance (< 4 Å) and favorable angles indicate a robust interaction, supporting metformin’s potential as a therapeutic modulator of the CDK4/cyclin D3 complex in cell cycle regulation.

Metformin docking with the Bcl-2 protein (PDB: 1G5M) reveals critical interactions within its functional domains, as illustrated in Fig. 10C. The ligand binds near the BH3 motif (residues 52–66) and adjacent regions, forming hydrogen bonds with Tyr28 (BH4 motif), Trp30 (BH3 motif), Ser167, and Asp171 (C-terminal). These interactions are stabilized by Van der Waals forces and aromatic stacking, particularly with Trp30 (Fig. 10C inset). Notably, the BH3 motif—central to pro-apoptotic activity—is directly engaged, suggesting metformin may disrupt Bcl-2’s anti-apoptotic function by interfering with its ability to bind pro-apoptotic BH3-only proteins (e.g., Bid, Bim).

The hydrogen bond between metformin and Ser167 (distance: 2.28 Å) implies perturbation of the C-terminal region, which modulates dimerization and membrane localization. Concurrently, interactions with Trp30 (2.02 Å) and Tyr28 (2.45 Å) likely destabilize the BH3-BH1 interface, a key hub for regulatory interactions. While the BH1 and BH2 motifs are structurally preserved, their functional crosstalk with the BH3 region is compromised, potentially impairing Bcl-2’s ability to inhibit apoptosis.

**Fig. 11:**
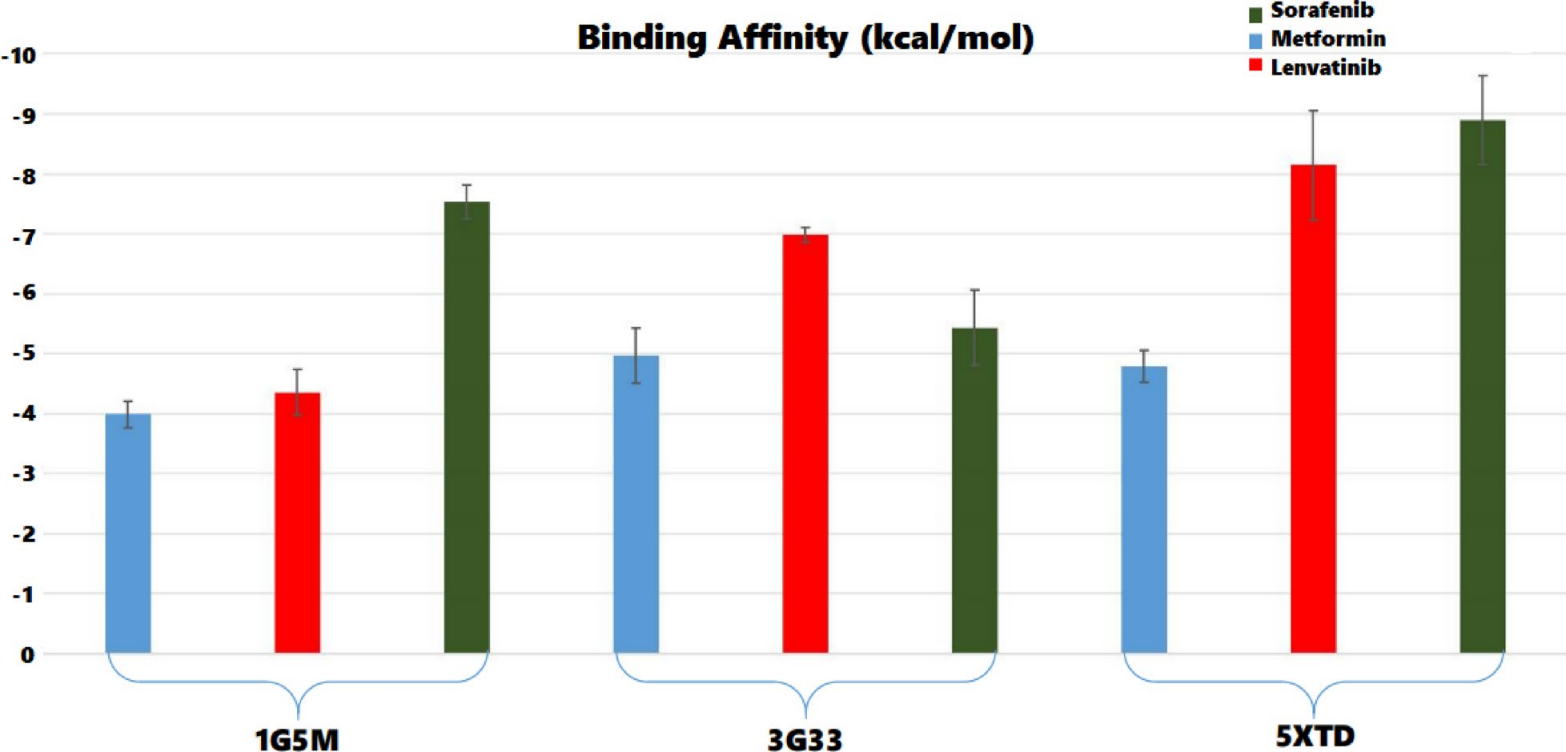
Illustrates the mean docking energies (kcal/mol) and standard deviations for metformin, lenvatinib, and sorafenib across three protein targets: Bcl-2 (1G5M), CDK4–cyclin D3 (3G33), and mitochondrial Complex I (5XTD). Bars represent the average of the top ten docking poses, with error bars indicating one standard deviation.

The docking analysis, based on the average of the top ten poses for each ligand–protein pair, revealed a broad spectrum of binding affinities across three target systems, as illustrated in Fig. 11. For the anti‑apoptotic Bcl‑2 protein (PDB ID: 1G5M), metformin exhibited the weakest interaction, with a mean absolute binding energy of 3.99 ± 0.22 kcal/mol, whereas lenvatinib and sorafenib showed substantially higher affinities (4.35 ± 0.38 kcal/mol and 7.54 ± 0.28 kcal/mol, respectively). In contrast, within the CDK4–cyclin D3 complex (PDB ID: 3G33), metformin’s mean score of 4.96 ± 0.46 kcal/mol indicates a moderate affinity relative to lenvatinib (6.98 ± 0.13 kcal/mol) and sorafenib (5.43 ± 0.63 kcal/mol). A similar trend was observed for mitochondrial Complex I (PDB ID: 5XTD), where metformin bound with a mean energy of 4.79 ± 0.27 kcal/mol compared to lenvatinib’s 8.15 ± 0.91 kcal/mol and sorafenib’s 8.89 ± 0.74 kcal/mol. Binding energies in the range of approximately 4–7 kcal/mol are generally considered indicative of moderate affinity^73^. This moderate binding by metformin to CDK4–cyclin D3 and Complex I suggests energetically feasible interactions that warrant further investigation. We subsequently performed molecular dynamics simulations to evaluate the stability of the docked complexes, systematically characterizing the temporal evolution of key hydrogen-bonding and hydrophobic interactions. Additionally, rigorous free energy calculations were conducted to assess the binding affinities. These analyses clarified that metformin can stably occupy the ATP-binding cleft of CDK4 and the ubiquinone-binding site of Complex I, supporting its potential role as a direct inhibitor or allosteric modulator within these targets.

#### Molecular dynamic simulation and MMPBSA analysis

##### Structural stability and interaction dynamics of the 3G33–metformin complex

**Fig. 12:**
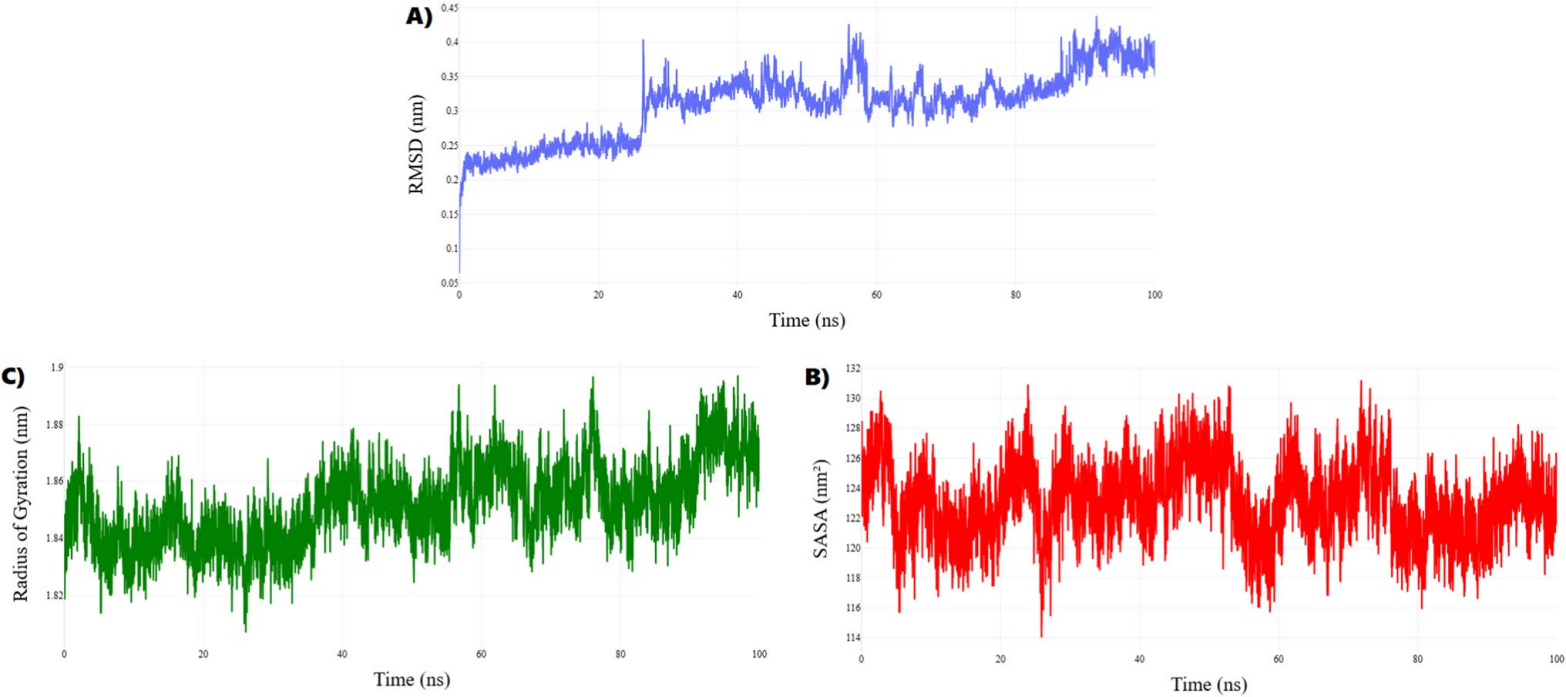
Molecular dynamics analysis of the 3G33–Metformin complex over 100 ns (**A**) RMSD reflects prolonged structural equilibration and sustained conformational shifts. (**B**) Rg indicates a maintained compact global fold. (**C**) SASA shows consistent solvent exposure, supporting global structural stability.

The structural dynamics of the 3G33–metformin complex were assessed through 100 ns molecular dynamics (MD) simulation. The root mean square deviation (RMSD) showed a rapid increase to ~ 0.23 nm within the first nanosecond, followed by a gradual rise to an average of 0.31 ± 0.04 nm over the final 90 ns, indicating slow, continuous conformational rearrangements without reaching full equilibrium (Fig. 12A). This trend suggests that metformin binding promotes long-timescale internal adjustments rather than immediate structural stabilization.

The radius of gyration (Rg) remained consistent, averaging 1.85 ± 0.01 nm, signifying that the protein maintained a compact and folded conformation throughout the simulation (Fig. 12B). Similarly, the solvent-accessible surface area (SASA) exhibited minimal fluctuation, with a stable mean of 122.5 ± 2.5 nm^2^ (Fig. 12C). Together, the stable Rg and SASA profiles indicate that the observed conformational changes are localized rather than global, preserving the protein’s overall structural integrity. These results imply that metformin engages the binding pocket without triggering large-scale unfolding or destabilization.

**Fig. 13:**
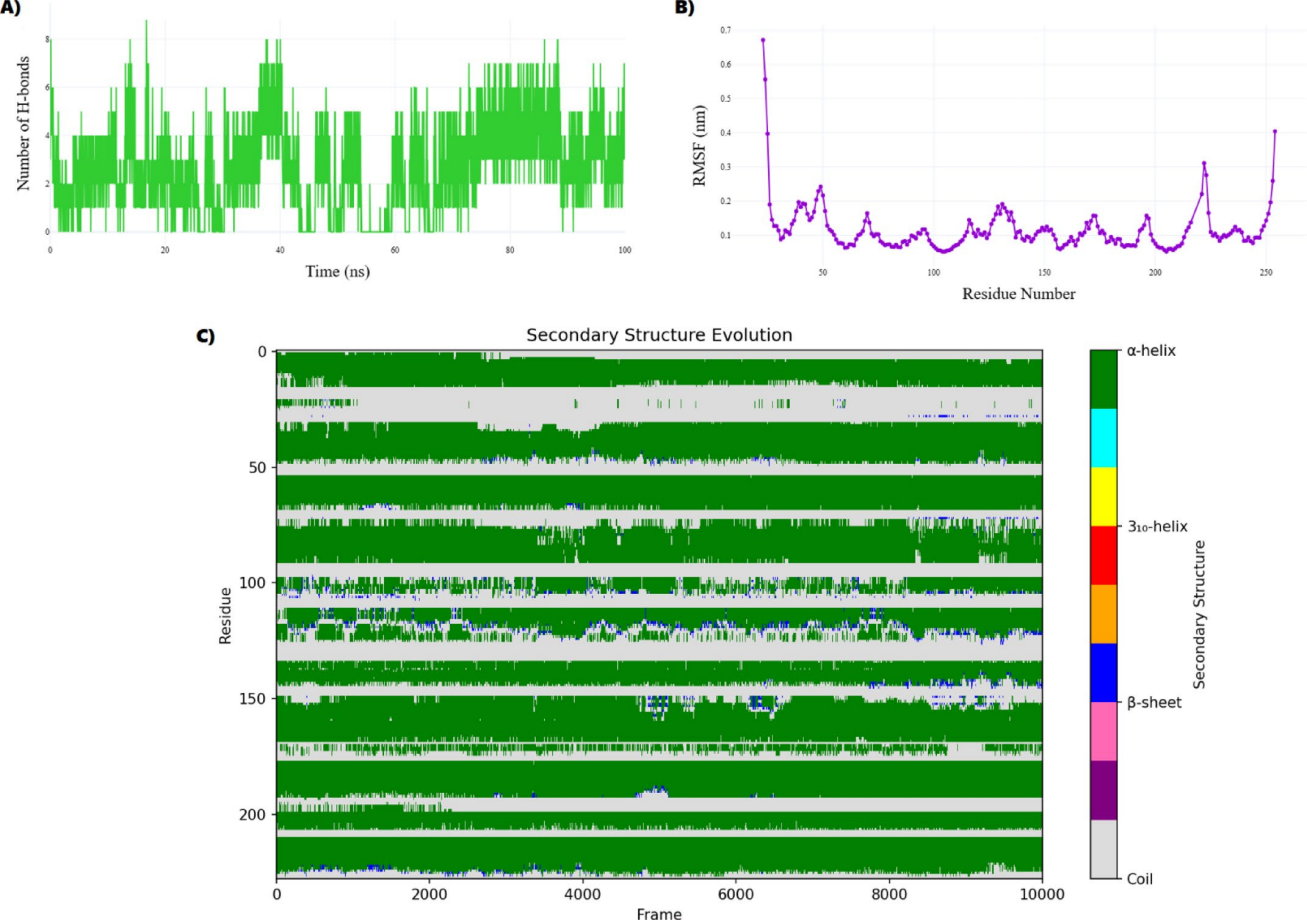
Local interaction dynamics and secondary structure disruption in the 3G33–Metformin complex. (**A**) Hydrogen bonding fluctuated between 0–9 bonds (avg. 2.61 ± 1.70), reflecting transient ligand engagement. (**B**) RMSF analysis identified flexible regions at residues 23–25 and stable core regions (e.g., residue 157), indicating localized mobility. (**C**) Secondary structure analysis revealed metformin-induced α-helix disruption, with 492 frames deviating beyond ± 2 SD. LEU145–LEU148 and ARG167 were hotspots of conformational instability.

In Fig. 13A illustrates the hydrogen bond dynamics between 3G33 and metformin, showing an average of 2.61 ± 1.70 hydrogen bonds and 2.13 ± 1.68 close contacts (within 0.35 nm) across the trajectory. The high standard deviations reflect dynamic fluctuations in these interactions, ranging from 0 to 9 hydrogen bonds, which are characteristic of aqueous environments and essential for maintaining reversible ligand binding and structural plasticity.

RMSF analysis (Fig. 13B) reveals an average atomic fluctuation of 0.11 ± 0.07 nm. Residues 23 (0.67 nm), 24 (0.56 nm), and 25 (0.40 nm) exhibited the highest mobility, likely corresponding to loop or unstructured regions facilitating ligand accommodation or allosteric regulation. In contrast, core residues such as 157 (0.0587 nm) displayed minimal fluctuations, indicative of structurally rigid domains that form the stable secondary structure core. These results underscore that while local flexibility supports dynamic ligand interactions, the overall structural coherence of the complex is preserved.

Despite the global structural stability observed in RMSD, Rg, and SASA metrics, secondary structure analysis (Fig. 13C) reveals significant localized fluctuations induced by metformin binding. The average α-helical content was 63.23% (SD = 2.44%), yet 492 frames deviated beyond ± 2 SD, indicating recurrent transitions between folded and partially unfolded conformations. These changes, while not affecting the protein’s compactness, point to a functionally relevant reorganization of secondary structural elements.

In particular, the segment LEU145–LEU148 exhibited persistent conformational variability, suggesting the presence of a flexible hinge region. ARG167 emerged as the most structurally labile residue, undergoing 3,688 transitions between defined secondary structure states, marking it as a hotspot of conformational flux. These dynamic features, although confined to specific residues, may play a critical regulatory role.

This localized structural volatility aligns with impaired CDK4 functionality. The LEU145–LEU148 segment likely acts as a destabilized helical junction, while ARG167’s instability may disrupt a conserved electrostatic interaction, similar to the Arg167-mediated clamp observed in the Hsp90–Cdc37–CDK4 complex. Given the significance of this clamp in stabilizing the CDK4 fold and its interactions with regulatory partners, metformin-induced disruption at this site could impair proper folding or substrate binding. Thus, while the protein retains its overall folded state, metformin binding introduces site-specific destabilization in key functional regions. These data support a mechanistic model in which metformin enforces G1/S arrest by structurally incapacitating CDK4 through targeted conformational destabilization, offering a quantitative basis for its antiproliferative activity.

##### MM-PBSA analysis of the 3G33–metformin complex

The MM-PBSA analysis of the metformin–3G33 (CDK4) complex, detailed in Table 5, demonstrates a highly favorable and spontaneous binding interaction, with a total binding free energy (ΔG) of − 27.33 kcal/mol. This affinity is overwhelmingly driven by electrostatic contributions (ΔEEL =  − 343.22 ± 28.32 kcal/mol), which significantly surpass the van der Waals component (ΔVDWAALS =  − 9.00 ± 3.50 kcal/mol). Although the polar solvation energy (ΔEGB =  + 327.86 ± 21.45 kcal/mol) exerts a strong opposing force, consistent with expected desolvation costs, the overall solvation penalty (ΔGSOLV =  + 324.89 kcal/mol) is marginally reduced by the non-polar term (ΔESURF =  − 2.97 ± 0.42 kcal/mol). The resulting gas-phase energy (ΔGGAS =  − 352.22 kcal/mol) reflects a robust interaction, with the relatively low standard deviations indicating minimal fluctuation in energetic stability throughout the simulation.

**Table 5:** MMPBSA energy components for the metformin–3G33 complex.

Energies (Kcal/mol)							
Frames	VDWAALS	EEL	EGB	ESURF	GGAS	GSOLV	Total
Average	− 9	− 343.22	327.86	− 2.97	− 352.22	324.89	− 27.33
SD	3.5	28.32	21.45	0.12	27.42	21.49	8.47
SEM	1.11	8.96	6.78	0.04	8.67	6.79	2.68

**Fig. 14:**
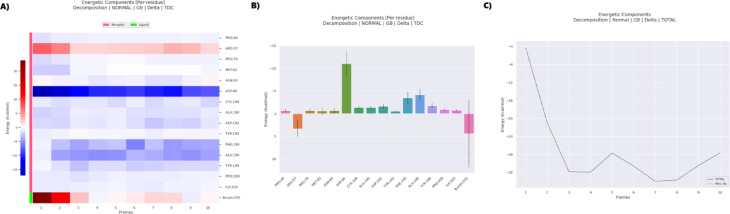
MMPBSA analysis and energetic decomposition of the 3G33–metformin complex (**A**) shows a heatmap of per-residue energy contributions, (**B**) displays the residue-wise energy distribution, and (**C**) depicts the temporal evolution of total binding energy over multiple simulation frames.

Energetic decomposition presented in Fig. 14 offers deeper insight into residue-specific contributions. As illustrated in Fig. 14A, a per-residue heatmap identifies ARG57 as a dominant binding hotspot, contributing a striking − 243.54 kcal/mol—suggestive of a persistent salt bridge with metformin. This is corroborated by the bar chart in Fig. 14B, which also highlights ASP86 (–101.64 kcal/mol), ASP192 (–91.04 kcal/mol), and ASN83 (–57.77 kcal/mol) as major contributors, reflecting the prevalence of strong electrostatic and hydrogen-bonding interactions. The time-resolved energy profile shown in Fig. 14C indicates stable binding dynamics, with only moderate fluctuations around the mean ΔG, reinforcing the conformational consistency of the ligand within the active site. Collectively, the data in Table 5 and Fig. 14 support a model wherein metformin occupies the ATP-binding pocket of CDK4, disrupting its kinase function by outcompeting ATP. This inhibitory mechanism is consistent with that of clinically used CDK4/6 inhibitors, reinforcing the potential of metformin as an anti-cancer agent through cell cycle arrest via blockade of Retinoblastoma protein phosphorylation.

##### Conformational flexibility and interaction dynamics of the 5XTD–metformin complex

**Fig. 15:**
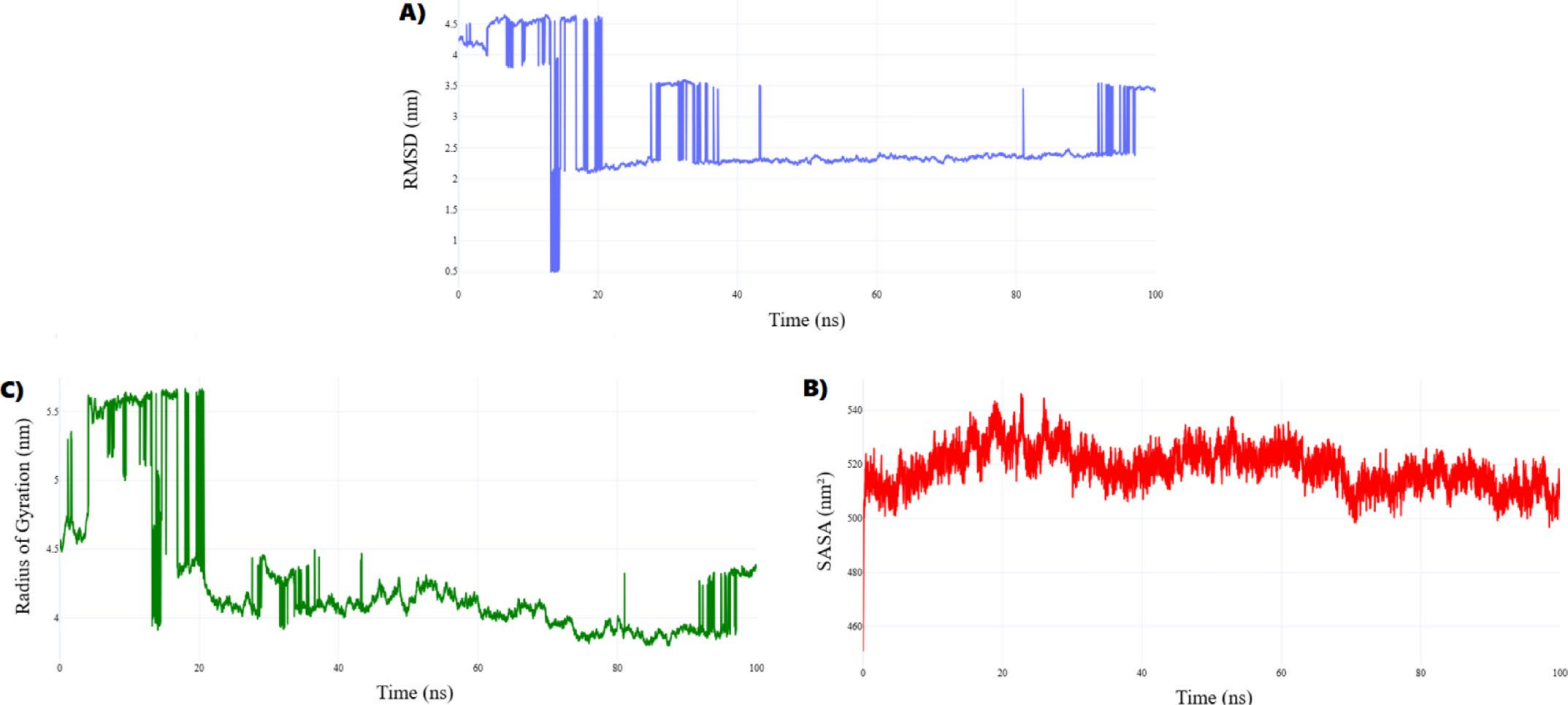
Global structural instability of the 5XTD–Metformin complex over 100 ns. (**A**) RMSD shows large deviations (avg. 3.56 ± 0.82 nm) with a spike to ~ 4.5 nm at 1.16 ns and sustained fluctuations, indicating conformational lability. (**B**) Radius of gyration increases (mean 4.65 ± 0.53 nm), with unfolding events at 1.16 and 1.6 ns, reflecting loss of compactness. (**C**) SASA (avg. 513.4 ± 8.2 nm^2^) shows transient rises, suggesting hydrophobic exposure and tertiary destabilization, particularly in NDUFV1.

Molecular dynamics simulations of the 5XTD–metformin complex over 100 ns revealed pronounced conformational plasticity, underscoring metformin’s role as a dynamic modulator of Complex I. Key structural parameters are summarized in Fig. 15A–C.

The RMSD profile (Fig. 15A) exhibited substantial deviations from the initial conformation (mean: 3.56 ± 0.82 nm), with a sharp spike at 1.16 ns (approximately 4.5 nm) and sustained fluctuations between 12 and 18 ns. These shifts reflect metformin-induced dynamic rearrangements, characteristic of reversible inhibition that perturbs Complex I structural stability.

The radius of gyration (Rg) (Fig. 15B), evaluated across the entire Complex I model, confirmed reduced compactness (mean: 4.65 ± 0.53 nm). Notable expansion phases at 1.16 ns and 1.6 ns aligned temporally with RMSD peaks, indicating global unfolding or subunit dislocation events. These disruptions may compromise supramolecular assembly, particularly affecting NDUFV1’s FMN-binding site critical for NADH oxidation.

The solvent-accessible surface area (SASA) (Fig. 15C), also computed for the full Complex I assembly, averaged 513.4 ± 8.2 nm^2^. Transient elevations coinciding with Rg and RMSD perturbations suggest exposure of typically buried domains. Notably, solvent exposure in the CHCH domain of NDUFV1—essential for redox function—supports tertiary structure destabilization upon metformin engagement.

**Fig. 16:**
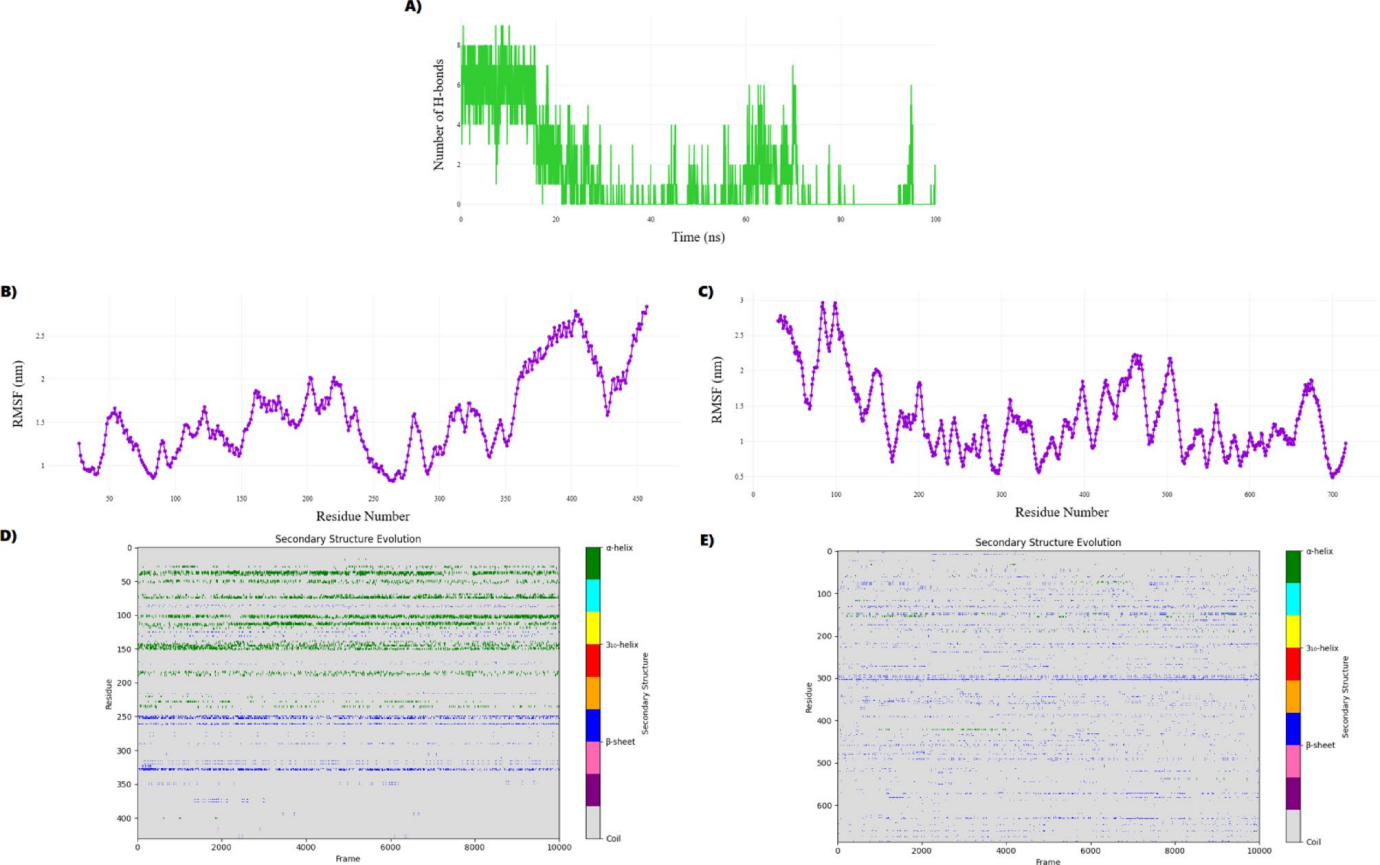
Metformin-induced conformational and dynamic perturbations in Complex I. (**A**) H-bond analysis shows stable binding to NDUFV1 (avg. 6 ± 2), with sporadic ND1 contacts. (**B**) RMSF of NDUFV1 highlights localized flexibility near FMN-binding loops (residues 45–60, 160–180). (**C**) ND1 exhibits high mobility at residues 80–100, peaking at 2.9 nm, indicating proton-pumping destabilization. (**D**) NDUFV1 secondary structure shifts show α-helix gain (48.2% ± 3.5%) in regions 43–54, 277–288, 343–356. (**E**) ND1 remains largely coil (97.52% ± 0.97%) but transiently forms β-sheets (max 5.24%) at Gly38–Tyr48, disrupting NADH binding.

Collectively, these metrics indicate that metformin disrupts Complex I structural integrity via increased flexibility, partial unfolding, and surface exposure. These conformational alterations weaken the architecture of both NDUFV1 and NADH-ubiquinone oxidoreductase chain 1 (ND1), as detailed in Fig. 16A–E, ultimately impairing catalytic performance and reducing ATP synthesis. This aligns with the mechanistic model of metformin as a reversible Complex I inhibitor that suppresses hepatic gluconeogenesis and alters mitochondrial bioenergetics^74–78^.

The hydrogen bonding analysis (Fig. 16A) revealed a dynamic interaction profile, with metformin forming an average of 6 ± 2 hydrogen bonds throughout the simulation. While NDUFV1 exhibited persistent interactions, ND1 (also known as NADH-ubiquinone oxidoreductase chain 1) formed sporadic, transient hydrogen bonds—rarely more than 1–2 and often absent altogether—suggesting ND1 is not a primary binding site. Instead, ND1 may be affected via allosteric destabilization.

RMSF analysis (Fig. 16B) showed that NDUFV1 retained overall structural rigidity, except in loop regions surrounding the FMN-binding site (residues 45–60 and 160–180), which showed increased fluctuations (approximately 1.7 nm). Such flexibility near the redox center may compromise electron transfer efficiency. In contrast, ND1 exhibited pronounced mobility (Fig. 16C), particularly in its N-terminal domain (residues 80–100), with peak fluctuations near residue 83 (approximately 2.9 nm). Given ND1’s role in proton translocation, this destabilization may uncouple redox activity from proton pumping—a hallmark function of Complex I. Taken together, Fig. 16A–C suggest that metformin exerts its inhibitory effect not by forming a static complex but through allosteric modulation that promotes conformational instability and hinders the coordinated function of catalytic and structural subunits.

In Fig. 16D, secondary structure analysis of NDUFV1 revealed metformin-induced stabilization of α-helical content (48.2% ± 3.5%), accompanied by reduced coil content (36.2% ± 4.2%) and relatively stable β-sheet levels (15.6% ± 2.1%). These changes were concentrated in residues 43–54, 277–288, and 343–356. For instance, TYR46 increased helical occupancy from 35 to 65%, and THR43 shifted from coil to 40% α-helix. Over 150 coil-to-helix transitions occurred within the 43–54 segment, a loop adjacent to the NADH-binding Rossmann fold. This structural rigidification may limit conformational adaptability required for efficient electron transfer.

In Fig. 16E, secondary structure transitions in ND1 were also evident. The chain was predominantly coil (mean: 97.52%, SD: 0.97%), with transient increases in β-sheet (mean: 1.51%, maximum: 5.24%) and α-helix (mean: 0.82%, maximum: 4.80%). The region spanning Gly38 to Tyr48 emerged as a conformational hotspot with substantial transitions from coil to β-sheet (8,332 transitions) and from β-sheet to coil (8,316 transitions). TYR47 and TYR48 had β-sheet occupancy greater than 16%, while GLY38 and GLY46 exceeded 7%. These residues are implicated in NADH binding and may influence the geometry of the ubiquinone channel, thus perturbing electron flux.

The combination of global and subunit-specific structural analyses presents a coherent picture of metformin as a reversible modulator of Complex I activity. Rather than acting as a static inhibitor, it exerts its effects through conformational destabilization and altered secondary structure dynamics, particularly near key functional domains in NDUFV1 and ND1. The Gly38 to Tyr48 motif within ND1 emerges as a structurally responsive region and may represent a viable target for the design of next-generation allosteric inhibitors.

##### MM-PBSA analysis of the 5XTD–metformin complex

The MM-PBSA analysis of the 5XTD–metformin complex, as summarized in Table 6, demonstrates that the ligand–receptor binding is primarily driven by electrostatic interactions, with an overall binding free energy (ΔTOTAL) of − 6.21 kcal/mol and a high standard deviation of ± 5.25 kcal/mol, suggesting substantial dynamic fluctuations throughout the simulation. This favorable binding is largely attributable to a strong electrostatic component (ΔEEL =  − 108.14 ± 20.91 kcal/mol), with minor support from van der Waals forces (ΔVDWAALS =  − 0.62 ± 3.94 kcal/mol), culminating in a notably negative gas-phase energy (ΔGGAS =  − 107.51 ± 17.67 kcal/mol). However, the gas-phase affinity is offset by a significant polar solvation penalty (ΔEGB =  + 102.48 ± 13.46 kcal/mol), with the total solvation energy (ΔGSOLV =  + 101.31 ± 13.39 kcal/mol) mitigated slightly by the nonpolar solvation term (ΔESURF =  − 1.17 kcal/mol).

**Table 6:** MMPBSA energy components for the metformin–5XTD complex.

Energies (kcal/mol)							
Frames	VDWAALS	EEL	EGB	ESURF	GGAS	GSOLV	Total
Average	0.62	− 108.14	102.48	− 1.17	− 107.51	101.31	− 6.21
SD	3.94	20.91	13.46	0.53	17.67	13.39	5.25
SEM	1.25	6.61	4.26	0.17	5.59	4.23	1.66

**Fig. 17:**
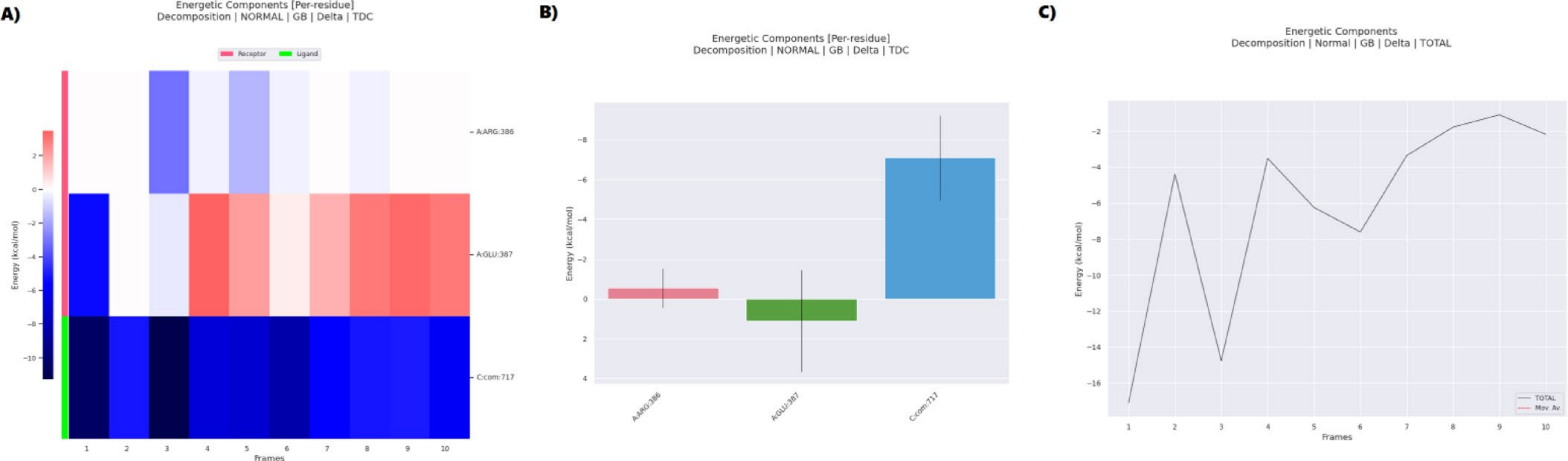
MMPBSA Analysis and Energetic Decomposition. (**A**) Heatmap of per-residue energy contributions highlighting dominant interactions. (**B**) Residue-wise energy distribution showing electrostatic (EEL) and solvation (EGB) contributions. (**C**) Temporal evolution of the total binding energy (black line, average =  − 6.21 kcal/mol) over simulation frames, with fluctuations (shaded area: ± 1 SD) reflecting dynamic stability. Key residues (ARG:386, GLU:387) drive binding through electrostatic and solvation effects.

Figure 17C illustrates the temporal progression of the binding energy, revealing pronounced fluctuations consistent with a metastable binding mode. The heatmap and decomposition plots in Fig. 17A,B, respectively, elucidate per-residue energy contributions. Notably, the ligand residue L:C:com:717 contributes − 7.10 kcal/mol, predominantly via electrostatic forces (ΔEEL =  − 54.07 kcal/mol), despite a counterbalancing polar solvation term (+ 47.42 kcal/mol). Within the receptor, ARG 386 is identified as a critical, yet energetically unstable residue, displaying strong but opposing electrostatic attraction (+ 16.79 kcal/mol) and solvation penalty (–17.49 kcal/mol), resulting in a near-neutral net effect (+ 0.55 kcal/mol, SD = 16.81 kcal/mol). Similarly, GLU 387 demonstrates intense attraction (ΔEEL =  − 43.68 kcal/mol) offset by solvation loss (+ 44.33 kcal/mol), yielding a marginal net gain (+ 1.10 kcal/mol, SD = 21.55 kcal/mol). THR 383 shows a minor stabilizing effect (–0.19 kcal/mol), indicating peripheral involvement.

## Conclusion

This work delivers an integrative preclinical evaluation of PEGylated liposomal metformin for hepatocellular carcinoma, combining physicochemical analyses, cellular assays, and computational modeling. We achieved high encapsulation efficiency (> 90%) and demonstrated that PEGylation modulates liposomal bilayer structure without compromising colloidal stability. In vitro, PEGylated formulations enhanced HepG2 cytotoxicity (IC₅₀ ≈ 119 µg/mL vs. 2393 µg/mL for free metformin) and promoted G₀/G₁ cell cycle arrest and apoptosis, although comparable toxicity in Vero cells at supratherapeutic doses indicates limited selectivity. Molecular docking and 100 ns MD/MM-PBSA simulations suggest feasible binding of metformin to mitochondrial Complex I and CDK4/cyclin D3, driven predominantly by electrostatic interactions (MM-PBSA ΔG =  − 27.33 kcal/mol for CDK4). Collectively, these data support the concept that liposomal delivery can improve metformin’s anticancer profile, but further in vivo validation is required before clinical translation.

## Limitations and future perspectives

Despite the encouraging results, this study has notable limitations that should guide future investigations. All biological assays were limited to a single hepatocellular carcinoma cell line (HepG2) and Vero kidney epithelial cells, which do not fully capture the genetic heterogeneity or tissue-specific environment of human liver cancer; therefore, additional experiments using multiple HCC cell lines, primary patient-derived cultures, or liver organoids are warranted to confirm reproducibility and assess off-target effects in healthy hepatic tissue. Furthermore, the observed particle size heterogeneity (TEM: 361 ± 150 nm) and relatively high polydispersity index (PDI = 0.61) could compromise tumor accumulation via the enhanced permeability and retention (EPR) effect, highlighting the need to refine formulation protocols for better size control and batch homogeneity. The absence of detailed drug-release profiles under tumor-like acidic or enzyme-rich conditions limits our understanding of the formulation’s potential for stimulus-responsive delivery. Additionally, while molecular docking and molecular dynamics/MM-PBSA simulations suggest plausible binding of metformin to mitochondrial Complex I and CDK4/cyclin D3, these computational findings require functional validation through target-specific biochemical assays, such as kinase inhibition or mitochondrial respiration measurements. Addressing these limitations through expanded in vitro studies, improved formulation techniques, release kinetics characterization, and mechanistic confirmation will be essential steps to advance PEGylated liposomal metformin toward robust preclinical and in vivo validation.

## Data Availability

The datasets generated and/or analyzed during the current study are not publicly available due to restrictions imposed by the institutional review board (IRB) to protect sensitive biological and experimental data, but they are available from the corresponding author upon reasonable request.

## Abbreviations

3D: Three-dimensional
ASGPR: Asialoglycoprotein receptor
CDK4: Cyclin-dependent kinase 4
Complex I: NADH:ubiquinone oxidoreductase (mitochondrial respiratory complex I)
DNA: Deoxyribonucleic acid
DLS: Dynamic light scattering
EPR: Enhanced permeability and retention
GMP: Good manufacturing practice
HCC: Hepatocellular carcinoma
IC₅₀: Half-maximal inhibitory concentration
ISO: International Organization for Standardization
MD: Molecular dynamics
MM-PBSA: Molecular mechanics Poisson–Boltzmann surface area
PDI: Polydispersity index
PEG: Polyethylene glycol
RNA: Ribonucleic acid
TEM: Transmission electron microscopy

## References

[1] Sung, H. et al. Global cancer statistics 2020: GLOBOCAN Estimates of incidence and mortality worldwide for 36 cancers in 185 countries. *CA Cancer J. Clin.* **71**(3), 209–249. 10.3322/caac.21660 (2021). 33538338 10.3322/caac.21660

[2] Llovet, J. M. et al. Hepatocellular carcinoma. *Nat. Rev. Dis. Primers* **7**(1), 6. 10.1038/s41572-020-00240-3 (2021). 33479224 10.1038/s41572-020-00240-3 PMC13537433

[3] Suresh, A. & Dhanasekaran, R. Implications of genetic heterogeneity in hepatocellular cancer. *Adv. Cancer Res.* **156**, 103–135. 10.1016/bs.acr.2022.01.007 (2022). 35961697 10.1016/bs.acr.2022.01.007 PMC10321863

[4] Reig, M. et al. BCLC strategy for prognosis prediction and treatment recommendation: The 2022 update. *J. Hepatol.* **76**(3), 681–693. 10.1016/j.jhep.2021.11.018 (2022). 34801630 10.1016/j.jhep.2021.11.018 PMC8866082

[5] Rimassa, L., Pressiani, T. & Merle, P. Systemic treatment options in hepatocellular carcinoma. *Liver Cancer* **8**(6), 427–446. 10.1159/000499765 (2019). 31799201 10.1159/000499765 PMC6883446

[6] Rena, G., Hardie, D. G. & Pearson, E. R. The mechanisms of action of metformin. *Diabetologia* **60**(9), 1577–1585. 10.1007/s00125-017-4342-z (2017). 28776086 10.1007/s00125-017-4342-z PMC5552828

[7] Christensen, M. M. et al. The pharmacogenetics of metformin and its impact on plasma metformin steady-state levels and glycosylated hemoglobin A1c. *Pharmacogenet. Genom.* **21**(12), 837–850. 10.1097/FPC.0b013e32834c0010 (2011). 10.1097/FPC.0b013e32834c0010 21989078

[8] Bridges, H. R. et al. Structural basis of mammalian respiratory complex I inhibition by medicinal biguanides. *Science* **379**(6630), 351–357. 10.1126/science.ade3332 (2023). 36701435 10.1126/science.ade3332 PMC7614227

[9] Foretz, M., Guigas, B. & Viollet, B. Metformin: Update on mechanisms of action and repurposing potential. *Nat. Rev. Endocrinol.* **19**(8), 460–476. 10.1038/s41574-023-00833-4 (2023). 37130947 10.1038/s41574-023-00833-4 PMC10153049

[10] Saxton, R. A. & Sabatini, D. M. mTOR signaling in growth, metabolism, and disease. *Cell* **168**(6), 960–976. 10.1016/j.cell.2017.02.004 (2017). 28283069 10.1016/j.cell.2017.02.004 PMC5394987

[11] Zhang, H., Gao, C., Fang, L., Zhao, H. C. & Yao, S. K. Metformin and reduced risk of hepatocellular carcinoma in diabetic patients: A meta-analysis. *Scand. J. Gastroenterol.* **48**(1), 78–87. 10.3109/00365521.2012.719926 (2013). 23137049 10.3109/00365521.2012.719926

[12] Gwak, H., Kim, Y., An, H., Dhanasekaran, D. N. & Song, Y. S. Metformin induces degradation of cyclin D1 via AMPK/GSK3β axis in ovarian cancer. *Mol. Carcinog.* **56**(2), 349–358. 10.1002/mc.22498 (2017). 27128966 10.1002/mc.22498

[13] Zhang, J. et al. Metformin regulates TET2 expression to inhibit endometrial carcinoma proliferation: A new mechanism. *Front. Oncol.* **12**, 856707. 10.3389/fonc.2022.856707 (2022). 35480097 10.3389/fonc.2022.856707 PMC9035737

[14] Deng, H. et al. SHMT inhibitor synergizes with 5-Fu to suppress gastric cancer via cell cycle arrest and chemoresistance alleviation. *npj Precis. Oncol.* **9**, 135. 10.1038/s41698-025-00926-5 (2025). 40346149 10.1038/s41698-025-00926-5 PMC12064653

[15] Chen, G., Nicula, D., Renko, K. & Derwahl, M. Synergistic anti-proliferative effect of metformin and sorafenib on growth of anaplastic thyroid cancer cells and their stem cells. *Oncol. Rep.* **33**(4), 1994–2000. 10.3892/or.2015.3805 (2015). 25683253 10.3892/or.2015.3805

[16] Gong, L., Goswami, S., Giacomini, K. M., Altman, R. B. & Klein, T. E. Metformin pathways: Pharmacokinetics and pharmacodynamics. *Pharmacogenet. Genom.* **22**(11), 820–827. 10.1097/FPC.0b013e3283559b22 (2012). 10.1097/FPC.0b013e3283559b22 PMC3651676 22722338

[17] Maeda, H. Toward a full understanding of the EPR effect in primary and metastatic tumors as well as issues related to its heterogeneity. *Adv. Drug Deliv. Rev.* **91**, 3–6. 10.1016/j.addr.2015.01.002 (2015). 25579058 10.1016/j.addr.2015.01.002

[18] Bulbake, U., Doppalapudi, S., Kommineni, N. & Khan, W. Liposomal formulations in clinical use: An updated review. *Pharmaceutics* **9**(2), 12. 10.3390/pharmaceutics9020012 (2017). 28346375 10.3390/pharmaceutics9020012 PMC5489929

[19] Allen, T. M. & Cullis, P. R. Liposomal drug delivery systems: From concept to clinical applications. *Adv. Drug Deliv. Rev.* **65**(1), 36–48. 10.1016/j.addr.2012.09.037 (2013). 23036225 10.1016/j.addr.2012.09.037

[20] Ammerman, N. C., Beier-Sexton, M. & Azad, A. F. Growth and maintenance of vero cell lines. *Curr. Protoc. Microbiol.*10.1002/9780471729259.mca04es11 (2008). 19016439 10.1002/9780471729259.mca04es11 PMC2657228

[21] Kotsari, M., Dimopoulou, V., Koskinas, J. & Armakolas, A. Immune system and hepatocellular carcinoma (HCC): New insights into HCC progression. *Int. J. Mol. Sci.* **24**(14), 11471. 10.3390/ijms241411471 (2023). 37511228 10.3390/ijms241411471 PMC10380581

[22] Bangham, A. D., Standish, M. M. & Watkins, J. C. Diffusion of univalent ions across the lamellae of swollen phospholipids. *J. Mol. Biol.* **13**(1), 238–252. 10.1016/s0022-2836(65)80093-6 (1965). 5859039 10.1016/s0022-2836(65)80093-6

[23] Chen, C. & Tripp, C. P. An infrared spectroscopic based method to measure membrane permeance in liposomes. *Biochem. Biophys. Acta.* **1778**(10), 2266–2272. 10.1016/j.bbamem.2008.05.010 (2008). 18571495 10.1016/j.bbamem.2008.05.010

[24] Bellamakondi, P. K. et al. In vitro cytotoxicity of Caralluma species by MTT and trypan blue dye exclusion. *Asian J. Pharm. Clin. Res.* **7**(2), 17–19 (2014).

[25] DeLean, A., Munson, P. J. & Rodbard, D. Simultaneous analysis of families of sigmoidal curves: Application to bioassay, radioligand assay, and physiological dose-response curves. *Am. J. Physiol.* **235**(2), E97–E102. 10.1152/ajpendo.1978.235.2.E97 (1978). 686171 10.1152/ajpendo.1978.235.2.E97

[26] Kutner, M. H., Nachtsheim, C. J., Neter, J. & Li, W. *Applied Linear Statistical Models* 5th edn. (McGraw-Hill, 2005).

[27] R Core Team (2024) R: A Language and Environment for Statistical Computing. R Foundation for Statistical Computing. https://www.R-project.org/

[28] Tukey, J. W. Comparing individual means in the analysis of variance. *Biometrics* **5**(2), 99–114 (1949). 18151955

[29] Singh, N. P., McCoy, M. T., Tice, R. R. & Schneider, E. L. A simple technique for quantitation of low levels of DNA damage in individual cells. *Exp. Cell Res.* **175**(1), 184–191. 10.1016/0014-4827(88)90265-0 (1988). 3345800 10.1016/0014-4827(88)90265-0

[30] Blasiak, J., Trzeciak, A. & Kowalik, J. Curcumin damages DNA in human gastric mucosa cells and lymphocytes. *J. Environ. Pathol. Toxicol. Oncol.* **18**(4), 271–276 (1999). 15281237

[31] Ghasemi, A. & Zahediasl, S. Normality tests for statistical analysis: A guide for non-statisticians. *Int. J. Endocrinol. Metab.* **10**(2), 486–489. 10.5812/ijem.3505 (2012). 23843808 10.5812/ijem.3505 PMC3693611

[32] Fu, L. et al. ADMETlab 3.0: An updated comprehensive online ADMET prediction platform enhanced with broader coverage, improved performance, API functionality and decision support. *Nucleic Acids Res.* **52**(W1), W422–W431. 10.1093/nar/gkae236 (2024). 38572755 10.1093/nar/gkae236 PMC11223840

[33] Jones, P. et al. InterProScan 5: Genome-scale protein function classification. *Bioinformatics (Oxford, England)* **30**(9), 1236–1240. 10.1093/bioinformatics/btu031 (2014). 24451626 10.1093/bioinformatics/btu031 PMC3998142

[34] Schrödinger, L., & DeLano, W. (2020). PyMOL. Retrieved from http://www.pymol.org/pymol

[35] McNutt, A. T. et al. GNINA 1.3: The next increment in molecular docking with deep learning. *J. Cheminform.* **17**, 28. 10.1186/s13321-025-00973-x (2025). 40025560 10.1186/s13321-025-00973-x PMC11874439

[36] Biovia, D. S. Discovery Studio Visualizer. San Diego (2019).

[37] Salentin, S., Schreiber, S., Haupt, V. J., Adasme, M. F. & Schroeder, M. PLIP: Fully automated protein-ligand interaction profiler. *Nucleic Acids Res.* **43**(W1), W443–W447. 10.1093/nar/gkv315 (2015). 25873628 10.1093/nar/gkv315 PMC4489249

[38] Abraham, M. J. et al. GROMACS: High performance molecular simulations through multi-level parallelism from laptops to supercomputers. *SoftwareX* **1–2**, 19–25. 10.1016/j.softx.2015.06.001 (2015).

[39] Hess, B., Kutzner, C., van der Spoel, D. & Lindahl, E. GROMACS 4: Algorithms for highly efficient, load-balanced, and scalable molecular simulation. *J. Chem. Theory Comput.* **4**(3), 435–447. 10.1021/ct700301q (2008). 26620784 10.1021/ct700301q

[40] Best, R. B. et al. Optimization of the additive CHARMM all-atom protein force field targeting improved sampling of the backbone φ, ψ and side-chain χ₁ and χ₂ dihedral angles. *J. Chem. Theory Comput.* **8**(9), 3257–3273. 10.1021/ct300400x (2012). 23341755 10.1021/ct300400x PMC3549273

[41] Vanommeslaeghe, K. et al. CHARMM general force field (CGenFF): A force field for drug-like molecules compatible with the CHARMM all-atom additive biological force fields. *J. Comput. Chem.* **31**(4), 671–690. 10.1002/jcc.21367 (2010). 19575467 10.1002/jcc.21367 PMC2888302

[42] Vanommeslaeghe, K. & MacKerell, A. D. Jr. Automation of the CHARMM General Force Field (CGenFF) I: Bond perception and atom typing. *J. Chem. Inf. Model.* **52**(12), 3144–3154. 10.1021/ci3003649 (2012). 23146088 10.1021/ci300363c PMC3528824

[43] Jorgensen, W. L., Chandrasekhar, J., Madura, J. D., Impey, R. W. & Klein, M. L. Comparison of simple potential functions for simulating liquid water. *J. Chem. Phys.* **79**(2), 926–935. 10.1063/1.445869 (1983).

[44] Bussi, G., Donadio, D. & Parrinello, M. Canonical sampling through velocity rescaling. *J. Chem. Phys.* **126**(1), 014101. 10.1063/1.2408420 (2007). 17212484 10.1063/1.2408420

[45] Parrinello, M. & Rahman, A. Polymorphic transitions in single crystals: A new molecular dynamics method. *J. Appl. Phys.* **52**(12), 7182–7190. 10.1063/1.328693 (1981).

[46] Darden, T., York, D. & Pedersen, L. Particle mesh Ewald: An N·log(N) method for Ewald sums in large systems. *J. Chem. Phys.* **98**(12), 10089–10092. 10.1063/1.464397 (1993).

[47] Abraham, M. J. et al. GROMACS: High performance molecular simulations through multi-level parallelism from laptops to supercomputers. *SoftwareX* **1–2**, 19–25. 10.1016/j.softx.2021.06.001 (2021).

[48] Kumari, R., Kumar, R., Open Source Drug Discovery Consortium, & Lynn, A. g_mmpbsa—A GROMACS tool for high-throughput MM-PBSA calculations. *J. Chem. Inf. and Model.* **54**(7), 1951–1962 (2014). 10.1021/ci500020m 10.1021/ci500020m 24850022

[49] Waskom, M. L. seaborn: Statistical data visualization. *J. Open Source Softw.* **6**(60), 3021 (2021). 10.21105/joss.03021

[50] Plotly Technologies Inc. (2015). Collaborative data science [Software]. Plotly Technologies Inc. Retrieved from https://plot.ly

[51] Hunter, J. D. Matplotlib: A 2D graphics environment. *Comput. Sci. Eng.* **9**(3), 90–95. 10.1109/MCSE.2007.55 (2007).

[52] Danaei, M. et al. Impact of particle size and polydispersity index on the clinical applications of lipidic nanocarrier systems. *Pharmaceutics* **10**(2), 57. 10.3390/pharmaceutics10020057 (2018). 29783687 10.3390/pharmaceutics10020057 PMC6027495

[53] Ruozi, B. et al. AFM, ESD, TEM, and CLSM in liposomal characterization: a comparative study. *Int. J. Nanomed.* **6**, 557–563. 10.2147/IJN.S14615 (2011). 10.2147/IJN.S14615 PMC3065801 21468358

[54] Severcan, F., Sahin, I. & Kazancı, N. Melatonin strongly interacts with zwitterionic model membranes—Evidence from Fourier transform infrared spectroscopy and differential scanning calorimetry. *Biochim. et Biophys. Acta BBA Biomembr.* **1668**(2), 215–222. 10.1016/j.bbamem.2005.03.003 (2005). 10.1016/j.bbamem.2004.12.009 15737332

[55] Franken, L. E., Boekema, E. J. & Stuart, M. C. A. Transmission electron microscopy as a tool for the characterization of soft materials: Application and interpretation. *Adv. Sci.* **4**(5), 1600476. 10.1002/advs.201600476 (2017). 10.1002/advs.201600476 PMC5441488 28546914

[56] Petrini, M. et al. Effects of surface charge, PEGylation and functionalization with dipalmitoylphosphatidyldiglycerol on liposome–cell interactions and local drug delivery to solid tumors via thermosensitive liposomes. *Int. J. Nanomed.* **16**, 4045–4061. 10.2147/IJN.S305106 (2021). 10.2147/IJN.S305106 PMC8214027 34163158

[57] Alshaer, W. et al. Quality by design approach in liposomal formulations: Robust product development. *Molecules (Basel, Switzerland)* **28**(1), 10. 10.3390/molecules28010010 (2022). 36615205 10.3390/molecules28010010 PMC9822211

[58] Tang, J. et al. Effect of size and pegylation of liposomes and peptide-based synthetic lipoproteins on tumor targeting. *Nanomed. Nanotechnol. Biol. Med.* **13**(6), 1869–1878. 10.1016/j.nano.2017.04.009 (2017). 10.1016/j.nano.2017.04.009 PMC5554706 28434931

[59] Németh, Z. et al. Quality by design-driven zeta potential optimisation study of liposomes with charge imparting membrane additives. *Pharmaceutics* **14**(9), 1798. 10.3390/pharmaceutics14091798 (2022). 36145546 10.3390/pharmaceutics14091798 PMC9503861

[60] Liu, P., Chen, G. & Zhang, J. A review of liposomes as a drug delivery system: current status of approved products, regulatory environments, and future perspectives. *Molecules (Basel, Switzerland)* **27**(4), 1372. 10.3390/molecules27041372 (2022). 35209162 10.3390/molecules27041372 PMC8879473

[61] Shafaa, M. W., Sabra, N. M. & Fouad, R. A. The extended ocular hypotensive effect of positive liposomal cholesterol bound timolol maleate in glaucomatous rabbits. *Biopharm. Drug Dispos.* **32**(9), 507–517. 10.1002/bdd.778 (2011). 22028305 10.1002/bdd.778

[62] Shafaa, M. W., Elshazly, A. H., Dakrory, A. Z. & Elsyed, M. R. Interaction of coenzyme Q10 with liposomes and its impact on suppression of selenite—Induced experimental cataract. *Adv. Pharm. Bull.* **8**(1), 1–9. 10.15171/apb.2018.001 (2018). 29670833 10.15171/apb.2018.001 PMC5896383

[63] Elkholy, N. S., Shafaa, M. W. & Mohammed, H. S. Biophysical characterization of lutein- or beta-carotene-loaded cationic liposomes. *RSC Adv.* **10**(54), 32409–32422. 10.1039/D0RA05683A (2020). 35685615 10.1039/d0ra05683a PMC9127840

[64] Koynova, R. & Caffrey, M. Phases and phase transitions of the phosphatidylcholines. *Biochim. et Biophys. Acta BBA Rev. Biomembr.* **1376**(1), 91–145. 10.1016/S0304-4157(98)00017-3 (1998). 10.1016/s0304-4157(98)00006-9 9666088

[65] Kolman, I., Pippa, N., Meristoudi, A., Pispas, S. & Demetzos, C. A dual-stimuli-responsive polymer into phospholipid membranes: A thermotropic approach. *J. Therm. Anal. Calorim.* **123**(3), 2257–2271. 10.1007/s10973-015-5080-4 (2016).

[66] Riske, K. A. et al. Lipid bilayer pre-transition as the beginning of the melting process. *Biochim. et Biophys. Acta BBA Biomembr.* **1788**(5), 954–963 (2009). 10.1016/j.bbamem.2009.01.007 19366598

[67] Bafna, S. S., Sun, T. & Baird, D. G. The role of partial miscibility on the properties of blends of a polyetherimide and two liquid crystalline polymers. *Polymer* **34**(4), 708–715. 10.1016/0032-3861(93)90089-8 (1993).

[68] Fa, N. et al. Effect of the antibiotic azithromycin on thermotropic behavior of DOPC or DPPC bilayers. *Chem. Phys. Lipid* **144**(1), 108–116. 10.1016/j.chemphyslip.2006.01.002 (2006). 10.1016/j.chemphyslip.2006.08.002 17007828

[69] Park, H. Y. et al. Induction of p53-independent apoptosis and G1 cell cycle arrest by fucoidan in hct116 human colorectal carcinoma cells. *Mar. Drugs* **15**(6), 154. 10.3390/md15060154 (2017). 28555064 10.3390/md15060154 PMC5484104

[70] Pires, D. E., Blundell, T. L. & Ascher, D. B. pkCSM: Predicting small-molecule pharmacokinetic and toxicity properties using graph-based signatures. *J. Med. Chem.* **58**(9), 4066–4072. 10.1021/acs.jmedchem.5b00104 (2015). 25860834 10.1021/acs.jmedchem.5b00104 PMC4434528

[71] Daina, A., Michielin, O. & Zoete, V. SwissADME: A free web tool to evaluate pharmacokinetics, drug-likeness and medicinal chemistry friendliness of small molecules. *Sci. Rep.* **7**, 42717. 10.1038/srep42717 (2017). 28256516 10.1038/srep42717 PMC5335600

[72] Chon, Y. E. et al. Sorafenib vs. Lenvatinib in advanced hepatocellular carcinoma after atezolizumab/bevacizumab failure: A real-world study. *Clin. Mol. Hepatol.* **30**(3), 345–359. 10.3350/cmh.2023.0553 (2024). 38468561 10.3350/cmh.2023.0553 PMC11261222

[73] Warren, G. L. et al. A critical assessment of docking programs and scoring functions. *J. Med. Chem.* **49**(20), 5912–5931. 10.1021/jm050362n (2006). 17004707 10.1021/jm050362n

[74] Andrzejewski, S. et al. Metformin directly acts on mitochondria to alter cellular bioenergetics. *Cancer Metab.* **2**(1), 1. 10.1186/2049-3002-2-12 (2014). 25184038 10.1186/2049-3002-2-12 PMC4147388

[75] Law, S. L., Lo, W. Y., Pai, S. H. & Teh, G. W. The electrokinetic behavior of liposomes adsorbed with bovine serum albumin. *Int. J. Pharm.* **43**(3), 257–260. 10.1016/0378-5173(88)90282-7 (1988).

[76] Makino, K. et al. Temperature- and ionic strength-induced conformational changes in the lipid head group region of liposomes as suggested by zeta potential data. *Biophys. Chem.* **41**(2), 175–183. 10.1016/0301-4622(91)80017-l (1991). 1773010 10.1016/0301-4622(91)80017-l

[77] Plank, L., Dahl, C. E. & Ware, B. R. Effect of sterol incorporation on head group separation in liposomes. *Chem. Phys. Lipid.* **36**(4), 319–328. 10.1016/0009-3084(85)90039-8 (1985). 10.1016/0009-3084(85)90039-8 3924423

[78] Sarkar, J., Singh, N., Meena, S. & Sinha, S. Staurosporine induces apoptosis in human papillomavirus positive oral cancer cells at G2/M phase by disrupting mitochondrial membrane potential and modulation of cell cytoskeleton. *Oral Oncol.* **45**(11), 974–979. 10.1016/j.oraloncology.2009.04.009 (2009). 19502099 10.1016/j.oraloncology.2009.04.009

