# Correction: PEGylated liposomal metformin overcomes pharmacokinetic barriers to trigger potent mitochondrial disruption and cell cycle arrest in hepatocellular carcinoma

**DOI:** 10.1038/s41598-025-25898-1

**Published:** 2025-10-28

**Authors:** Zeinab A. Elzanaty, Medhat W. Shafaa, Seifeldin Elabed, Mohamed M. Omran

**Affiliations:** 1 Biochemistry Division, Chemistry Department, Faculty of Science, Helwan University, Cairo, Egypt; 2 Medical Biophysics Division, Physics Department, Faculty of Science, Helwan University, Cairo, Egypt

Correction to: *Scientific Reports* 10.1038/s41598-025-13280-0, published online 07 August 2025

The original version of this Article contained an error in the Figures, where Figure 16 was a duplication of Figure 13.

The original Figure 16 and accompanying legend appear below.

**Fig. 16:**
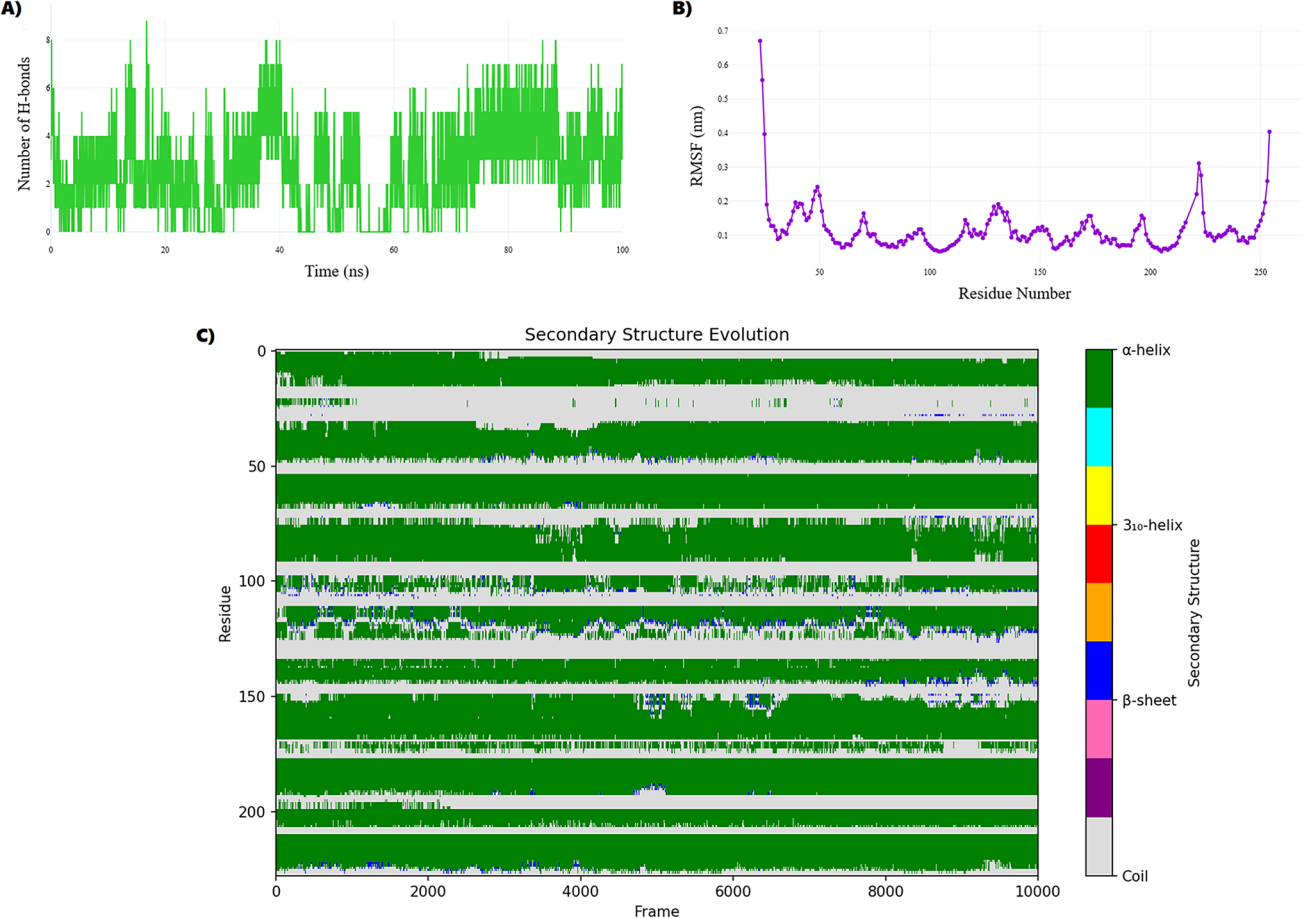
Metformin-induced conformational and dynamic perturbations in Complex I. (**A**) H-bond analysis shows stable binding to NDUFV1 (avg. 6 ± 2), with sporadic ND1 contacts. (**B**) RMSF of NDUFV1 highlights localized flexibility near FMN-binding loops (residues 45–60, 160–180). (**C**) ND1 exhibits high mobility at residues 80–100, peaking at 2.9 nm, indicating proton-pumping destabilization. (**D**) NDUFV1 secondary structure shifts show α-helix gain (48.2% ± 3.5%) in regions 43–54, 277–288, 343–356. (**E**) ND1 remains largely coil (97.52% ± 0.97%) but transiently forms β-sheets (max 5.24%) at Gly38–Tyr48, disrupting NADH binding.

The original Article has been corrected.

